# The genome of *Prunus humilis* provides new insights to drought adaption and population diversity

**DOI:** 10.1093/dnares/dsac021

**Published:** 2022-06-25

**Authors:** Yi Wang, Jun Xie, HongNa Zhang, Weidong Li, Zhanjun Wang, Huayang Li, Qian Tong, Gaixia Qiao, Yujuan Liu, Ying Tian, Yong Zan Wei, Ping Li, Rong Wang, Weiping Cheng, Zhengchang Liang, Meilong Xu

**Affiliations:** Institute of Horticulture, Ningxia Academy of Agricultural and Forestry Sciences, Yinchuan, 750012, China; Beijing Key Laboratory of Grape Sciences and Enology, Laboratory of Plant Resources, Institute of Botany, the Chinese Academy of Sciences, Beijing, 100093, China; State Key Laboratory of the Seedling Bioengineering, Yinchuan, 750004, China; Hainan University, Haikou 570228, China; School of Chinese Materia Medica, Beijing University of Chinese Medicine, Beijing 102488, China; Institute of Desertification Control, Ningxia Academy of Agricultural and Forestry Sciences, Yinchuan, 750012, China; Beijing Key Laboratory of Grape Sciences and Enology, Laboratory of Plant Resources, Institute of Botany, the Chinese Academy of Sciences, Beijing, 100093, China; Beijing Key Laboratory of Grape Sciences and Enology, Laboratory of Plant Resources, Institute of Botany, the Chinese Academy of Sciences, Beijing, 100093, China; State Key Laboratory of the Seedling Bioengineering, Yinchuan, 750004, China; State Key Laboratory of the Seedling Bioengineering, Yinchuan, 750004, China; State Key Laboratory of the Seedling Bioengineering, Yinchuan, 750004, China; Key Laboratory of Biology and Genetic Resources of Tropical Crops, Ministry of Agriculture, Institute of Tropical Bioscience and Biotechnology, Chinese Academy of Tropical Agricultural Sciences, Haikou, 571101, China; School of Chinese Materia Medica, Beijing University of Chinese Medicine, Beijing 102488, China; State Key Laboratory of the Seedling Bioengineering, Yinchuan, 750004, China; Institute of Horticulture, Ningxia Academy of Agricultural and Forestry Sciences, Yinchuan, 750012, China; Beijing Key Laboratory of Grape Sciences and Enology, Laboratory of Plant Resources, Institute of Botany, the Chinese Academy of Sciences, Beijing, 100093, China; Institute of Horticulture, Ningxia Academy of Agricultural and Forestry Sciences, Yinchuan, 750012, China

**Keywords:** *P. humilis*, genome, drought adaption, population diversity

## Abstract

*Prunus humilis* (2*n* = 2*x* = 16) is a dwarf shrub fruit tree native to China and distributed widely in the cold and arid northern region. In this study, we obtained the whole genome sequences of *P. humilis* by combining Illumina, PacBio and HiC sequencing technologies. This genome was 254.38 Mb long and encodes 28,301 putative proteins. Phylogenetic analysis indicated that *P. humilis* shares the same ancestor with *Prunus mume* and *Prunus armeniaca* at ∼ 29.03 Mya. Gene expansion analysis implied that the expansion of *WAX-related* and *LEA* genes might be associated with high drought tolerance of *P. humilis* and LTR maybe one of the driver factors for the drought adaption by increase the copy number of *LEAs.* Population diversity analysis among 20 *P. humilis* accessions found that the genetic diversity of *P. humilis* populations was limited, only 1.40% base pairs were different with each other, and more wild resources need to be collected and utilized in the breeding and improvement. This study provides new insights to the drought adaption and population diversity of *P. humilis* that could be used as a potential model plant for horticultural research.

## 1. Introduction

*Prunus humilis* Beg. (2*n* = 2*x* = 16), a member of the genus *Prunus*, is a dwarf shrub fruit tree native to China that is considered to be originated in the northern China. It is distributed in 14 provinces in northern China, mainly concentrated in the Zhongtiao Mountain and Taihang Mountain of Shanxi province, Daqing Mountain, Manhan Mountain and Horqin Grassland of Inner Mongolia.^1^ These habitats required *P. humilis* to be saline-, alkali- and cold-resistant, especially drought-resistant.^2^ It can grow normally at average annual rainfall of 80 mm.^3^ Root–shoot ratio of *P. humilis* was 9:1, and the ratio was seven times of *M. domestica* and 1.6 times of *P. armeniaca*. Which indicated *P. humilis* had better adaptability under drought stress partly because of well-developed root system.^4^

*Prunus*  *humilis* fruits contain sugar, rich amino acids, vitamin C and mineral nutrients. The Ca and Fe content is higher than many fruits.^5–7^ The fruits of *P. humilis* are consumed fresh or processed, and also used as the raw materials for wine, jam, vinegar and other processed products. In addition, the fruit kernel of *P. humilis*, called ‘Yuliren’ in China, is used for medicine for over 2000 years.^8^ Therefore, *P. humilis* is now planted in a large area in northern China that has great potential for becoming a horticulturally important fruit tree.

The current research on *P. humilis* in China mainly focus on the exploitation of wild germplasm resources, breeding new varieties and developing processed products. Few studies are conducted to understand genomics and functional genomics of *P. humilis* in its adaptation and tolerance to abiotic stresses.

In the present study, we analysed genomics of *P. humilis* and its phylogenetic relationship to related plant species. The results provide new insights on the molecular mechanism of high drought tolerance. The genomic resources also help accelerate the breeding of new *P. humilis* cultivars.

## 2. Materials and methods

### 2.1. Plant materials

Young leaves of five wide cultivated *P. humilis* varieties were collected for genome survey, and ‘Jing ou No.2’ (JO-2) was selected for genome sequencing. Young leaves, stems and green fruits of ‘Jing ou No.2’ were used for RNA isolation and tissue-specific transcriptome analysis. For identification and analysis of high drought tolerance-related genes of *P. humilis*, 1-year-old ‘Jing ou No.2’ plantlets were grown in a greenhouse under 40–50% of field capacity in 15 × 20 cm pots for 1 week. The same plantlets under 80–90% of field capacity were used as control. Three biological replicates were collected for each sample and immediately frozen in liquid nitrogen. Samples were stored at –80°C for RNA sequencing.

### 2.2. Genome sequencing

Genomic DNA of *P. humilis* was extracted from young leaves using CTAB protocol with slightly modifications. The extracted and concentrated DNA was used to generate sequences following standard protocols with both PacBio Sequel sequencer (Pacific Biosciences of California, Menlo Park, CA, USA) and Illumina Hiseq (Illumina, San Diego, CA, USA) platforms at Personal Biotechnology Co., Ltd (Shanghai, China); 450-bp insertions libraries were prepared for Illumina Hiseq platform and 150-bp paired-end reads were obtained.

### 2.3. Estimation of genome size

The evaluation of the genome size of *P. humilis* was performed using *K-mer* analysis based on the paired-end reads. Jellyfish (v2.2.6)^9^ with a *K-mer* of 19, genome size = total K-mer/K-mer depth was used to estimate the genome size. Genomic heterozygosity rate was estimated by the online tool GenomeScope^10^ (v2.0) (http://qb.cshl.edu/genomescope/).

### 2.4. Genome assembly

The raw PacBio data were corrected, trimmed and assembled by CANU-1.8.^11^ All Illumina and PacBio reads were mapped onto the assembled sequences to polish the assembly using Pilon (v1.22)^12^ for three rounds. Finally, the redundancy of polished sequence was removed by Redundans and Purge Haplotigs.^13^

### 2.5. Hi-C library construction and pseudo-chromosome construction

Young leaves of *P. humilis* were collected as the sample for Hi-C library construction, the pre-treatment of the sample was performed as described by Wang et al.^14^ with DpnII restriction enzyme, Hi-C libraries were sequenced by Illumina HiSeq with PE150 platform. The clean Hi-C reads were mapped onto the polished contigs using Juicer.^15^ And then, 3d-DNA^16^ was used to construct the genome sequence with the detail parameter ‘-i 15000 –editor-saturation-centile 10 –editor-coarse-resolution 100000 –editor-coarse-region 400000 –editor-repeat-coverage 100’.

### 2.6. Genome assembly evaluation

In this study, the assembled *P. humilis* genome was evaluated by calculating the homozygous mutation and benchmarking universal single-copy orthologs (BUSCOs). Approximately 23 Gb whole genome sequence (WGS) paired-end reads were mapped onto the assembled *P. humilis* genome, and only the homozygous mutations were calculated as the error rate. The assembled genome were also identified by BUSCOv2^17^ based on Embryophyta gene pool of eudicotyledons odb10.

### 2.7. Gene annotation

The repeat library of *P. humilis* was generated with RepeatModeler (v1.0.11, http://www.repeatmasker.org/RepeatModeler/) and the repeat annotation was processed with RepeatMasker (v4.0.7, http://www.repeatmasker.org/) based on Repbase^18^ (v20170127, https://www.girinst.org/) and Dfam^19^ (v20170127, http://www.dfam.org/). Simple sequence repeat (SSRs) were identified using MISA (http://pgrc.ipk-gatersleben.de/misa/misa.html) with the unit lengths ranging from 1 to 7 and the min-length was set to 10 bp. LTR were identified and analysed by LTR_retriever following the method of Ou and Jiang.^20^

### 2.8. Gene model prediction

A comprehensive strategy combining transcriptome-based, *ab initio* and homology-based approaches were combined to annotate protein-coding genes. First, AUGUSTUS (v3.3),^21^ SNAP^22^ and GeneMarkHMM (v 4.32)^23^ with default parameters were employed for protein-coding gene prediction in the *P. humilis* genome assembly. Second, RNA-seq data from leaf, steam and fruit of *P. humilis* were assembled by Trinity^24^ (v2.2.0), and then transcripts were aligned to the assembled genome for predicting ORFs by PASA^25^ (v2.0.2). Third, the protein homologues annotation was performed by GenomeThreader^26^ based on the proteins from *Malus pumila*, *P.*  *armeniaca*, *Amygdalus persica*, *Rosa chinensis* and many NCBI proteins belongs to Rosaceae. Finally, all the genes predicted by the above three approaches were integrated into the final annotation file using EVM^27^ (v 1.1.1), and the predicated genes were renamed according to their order in the genome sequence with the prefix of CH.

### 2.9. Treatment of WGS data

In this study, 20 core germplasms of *P. humilis* were re-sequenced following standard protocols with Illumina Hiseq 2500 (Illumina, San Diego, CA, USA) platforms at Personal Biotechnology Co., Ltd (Shanghai, China). The quality control was processed by Trimmomatic^28^ (v0.36) with shortest reads length of 90 bp, and the resulting data were mapped to the assembled genome by BWA^29^ (0.7.15). The mapped files were sorted and treated using Picard (v1.117, http://broadinstitute.github.io/picard/) and GATK^30^ (v3.5), and the SNPs were obtained using the UnifiedGenotyper function of GATK. Finally, the obtained SNPs were filtered by miss rate < 10% and minor allele frequency(MAF) > 5%.

### 2.10. Phylogenomic tree construction and global gene family analyses

We used OrthoMCL^31^ (v1.1.4) to identify super gene families based on the gene cluster of the 10 species, including *P. humilis*, *P. mume*, *P. armeniaca*, *P. persica*, *C.*  *pseudocerasus*, *M. domestica*, *Pyrus*  *bretschneideri*, *Rubus*  *corchorifolius*, *R. chinensis*, *Fragaria*  *ananassa* and *Vitis*  *vinifera* were used as outgroup in this study. Self BLASTP^32^ of all protein sequences were processed with an e-value cutoff of 1e-05 and OrthoMCL were used to cluster the orthologous genes. Single-copy genes were selected from the clustering results with all members and only one copy existed. Multiple alignment of the single copy protein sequences was performed by MUSCLE^33^ (v3.8.31). The phylogenetic tree were constructed by RAxML^34^ (v2.5.1) with the model of PROTGAMMAJTTF. The phylogenetic tree of 20 accessions of *P. humilis* were constructed by FastTree based on 4.91 Mb high quality SNPs of the population.

The divergence time between different species was calculated by MCMCtree (4.8a) from the PAML package^35^ based on four calibrations (*P. persica* vs *P. mume*, *C. pseudocerasus* vs *P. persica*, *M.*  *domestica* vs *P. bretschneideri*, *R.*  *chinensis* vs *F. ananassa*) from TimeTree^36^ (http://www.timetree.org). CAFÉ^37^ (v3.1) was used to estimate the gene expansion based on the OrthMCL results and times divergence tree.

### 2.11. Synteny analyses

Syntenic blocks between *P.*  *humilis*, *P. mume* and *P. persica* were identified by MCscanX, and blocks larger than 10 genes were obtained and exhibited by CIRCOS (0.67).^38^ The synonymous nucleotide change rate (Ks) between pairs of orthologous genes between any two genomes of *P.*  *humilis*, *P. mume* and *P. persica* genomes were calculated by KaKs Calculator (v 2.0),^39^ with the methods of NG. The synteny map between three species was generated by a python scripts contained in MCscan packages.

### 2.12. Drought stress treatment

The annual *P. humilis* seedlings with uniform growth were selected as drought stress treatment materials. First, the selected seedlings were transplanted into the round pots with a diameter of 150 mm for 6 months. Second, weight was used to calculate the field capacity of the soil. The drought stress treatment was continued for 14 days with 40–50% of field capacity, and 80–90% of field capacity was used as control. There were three biological replicates in different treatments.

### 2.13. RNA extraction and transcriptome analysis

Leaves, berry and stem of *P.*  *humilis* were sampled for RNA extraction. Total RNA was extracted using TRIzol Reagent (Plant RNA Purification Reagent for plant tissue) following the manufacturer’s instructions (Invitrogen, Carlsbad, CA, USA), and RNA quality was determined by 2100 Bioanalyzer (Agilent, Santa Clara, CA, USA) and quantified using the ND-2000 (NanoDrop Technologies, Wilmington, DE, USA). Only high-quality RNA sample (RIN ≥ 6.5) was used to construct sequencing library. The NEB Next Ultra Directional RNA Library Prep Kit for Illumina (New England BioLabs) was used to prepare the strand-specific mRNA libraries, and the libraries were sequenced on the Hiseq platform (Illumina) with the paired-end 150 bp sequencing mode at Personal Biotechnology Co., Ltd (Shanghai, China). Libraries derived from drought treated and materials at different field capacity were sequenced using the Hiseq 2000 platform in the single end 100 bp mode.

Trimmomatic^38^ (v0.36) was used to trim the sequences with the least length of 90 bp. All clean reads were mapped to the assembled *P. humilis* genome using Tophat2^40^ (v2.1.1). Gene expression levels were performed using Cufflinks^41^ (v2.2.1), and Cuffdiff^38^ was used to identify differentially expressed genes (DEGs) with a false discovery rate (FDR) < 0.05 and estimated absolute log_2_（LFC）> 1.

## 3. Results

### 3.1. Genome survey of the *P. humilis* population

In this study, genome size of five widely cultivated varieties (JO-1, JO-2, ND-4, ND-5, ND-6) were surveyed (Supplementary Table S1). JO-2 was selected for its lower heterozygosity and wide cultivation. The result indicated that the genome size of JO-2 is ∼ 247.18 Mb, and the heterozygosity is ∼ 0.70% (Supplementary Fig. S1).

### 3.2. Genome sequence and assembly

**Figure 1:**
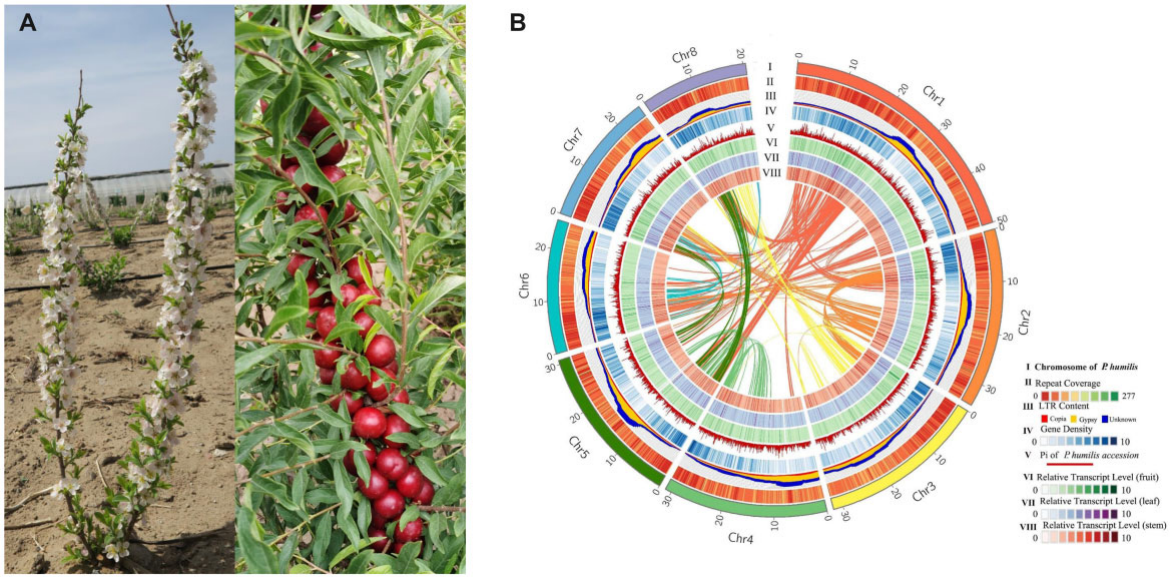
Growth condition, flowers and fruits, and genome landscape of *P. humilis*. (A) The blooming flowers and ripening fruits of *P. humilis* in the field; (B) The landscape of *P. humilis* genome. I the eight chromosomes of *P. humilis*; II the distribution of the repeat sequence on the genome of *P. humilis*; III LTR content on the genome of *P. humilis*. IV gene density distribution on the whole genome. V the diversity of populations of 20 *P. humilis* accessions. VI, VII and VIII the relative expression level of genes in fruit, leaf and stem of *P. humilis*, respectively.

In this study, 23.34 Gb Illumina Hiseq pair end short reads, 27.49 Gb PacBio Sequel single molecular long reads (2,334,777 sub-reads, N50 = 14.94 Kb) and 126.64 Gb Hi-C data (Supplementary Table S2) were combined to assemble the genome sequence. The correction and assemble of sub-reads were all processed by CANU-1.8. The PacBio long reads were first corrected and 9.31 Gb corrected reads were obtained with the N50 of 22.19 Kb (Supplementary Table S3). The corrected reads were then trimmed and retained 7.48 Gb high quality sub-reads (428,930 sub-reads, N50 = 18.79 Kb). Finally, we obtained 256.65 Mb contigs and the N50 of these contigs were 381.96 Kb (Supplementary Table S3). After redundancy removal by Purge Haplotigs, these contigs were assembled into scaffolds by Hi-C, finally we obtained 143 scaffolds containing 8 chromosome-level scaffolds with N50 of 31.26 Mb and genome size of 254.38 Mb (Supplementary Fig. S2), and 98.06% sequences were anchored onto the chromosomes. The chromosomes were named from Chr1 to Chr8 according to their chromosome size (Fig. 1).

The quality of this assembly was verified by three methods. First, ∼90X Illumina short reads were mapped onto the assembled genome and 99.53% reads were mapped properly. According to this mapping, only 19,528 homozygous SNPs and InDels were identified among the whole genome, indicating 0.0076% error rate or 13.026 Kb per error. Second, according to the BUSCO analysis, 97.3% of 2121 eudicotyledons single copy genes could be found in *P. humilis* genome, and 96.3% of them were complete genes (Supplementary Table S4). Finally, we also verified the assembly quality by RNA-seq from three different tissues (leaf, fruit and stem) of *P. humilis*. The result showed that the mapping rate of these RNA-seq data was ranged from 93.4% to 95.1% (Supplementary Table S5). These results all implied that this assembly generated a high-quality genome.

### 3.3. Element identification and genome annotation

In total, 93,485,481 bp (36.75%) sequence of the whole genome were identified as repetitive sequences. Among these repetitive sequences, LTR elements contributed 18.98% (48,270,572 bp), and DNA elements occupied 11.90% (30,261,512 bp) of the whole genome (Supplementary Table S6). LTR analysis showed that more than 70% (22,968,169 bp) of the LTRs were unknown types, and LTR/Gypsy occupied ∼ 19.0% (19,707,344 bp), the rest 10.66% (5,843,924 bp) were LTR/Copia (Supplementary Table S7).

The gene model annotation of *P. humilis* combined transcript-based, homology-based and *ab initio* prediction. The integrated results showed that there were 28,301 protein-coding genes in the whole genome, smaller than *P.*  *mume* (31,390) and *P.*  *armeniaca* (30,436). Among all these genes, 27,932 (98.70%) of them were anchored on the chromosomes. Beside the protein-coding genes, we also identified 1671 small RNAs in this genome, including 872 tRNAs, 250 rRNAs, 142 microRNAs and 407 snoRNAs (Supplementary Fig. S3). These small RNAs distributed uniformly on the whole genome except rRNAs, there exist three rRNAs enrichment regions on Chr2, Chr4 and Chr6 (Supplementary Fig. S4).

In addition, protein-coding genes were functionally annotated by NCBI, Swissprot, Pfam, GO and KEGG database; 26,509 (93.67%) genes can be annotated properly by at least one database (Supplementary Fig. S5).

### 3.4. Evolution history and comparative analysis of *P. humilis*

**Figure 2:**
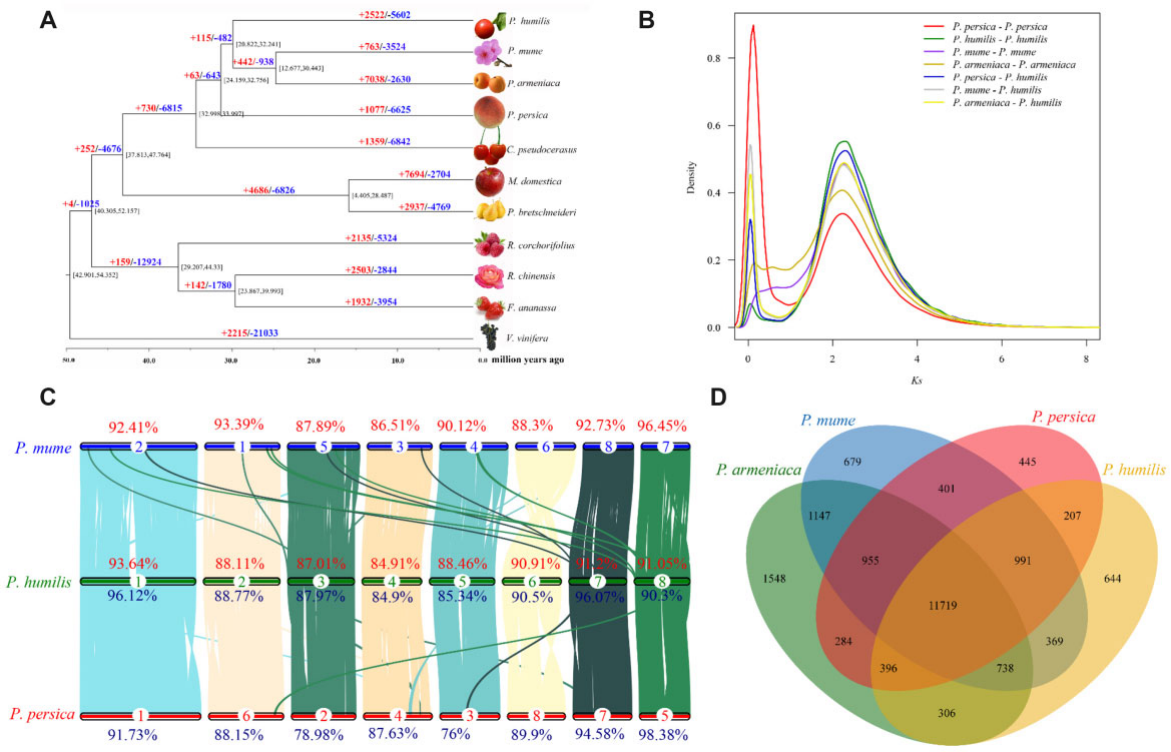
Phylogenetic and comparative genome analysis of *P. humilis*. (A) Phylogenetic relationship of 10 Rosaceae species with *V. vinifera* as outgroup. (B) *Ks* distribution of WGD (spell WGD out) and divergence for *P. humilis, P. persica, P. mume* and *P. armeniaca.* (C) Synteny analysis between *P. humilis, P. persica* and *P. mume.* (D) The overlap of specific genes between *P. humilis, P. persica, P. mume* and *P. armeniaca*.

In this study, 10 Rosaceae plant genomes and an out-group (*V.*  *vinifera*) were used to study the evolutionary position of *P. humilis*. Among these 11 plant species, 33,254 orthologous families and 1,552 single copy families were identified. Based on these single copy genes, the phylogenetic relationships among 10 Rosaceae species were constructed (Fig. 2A). According to this phylogenetic tree, *P. humilis* belongs to *Prunus* genus, and has close genetic relationship with *P. armeniaca* and *P. mume* (Fig. 2A, Supplementary Fig. S6). According to the molecular clock analysis, *P. humilis*, *P. mume* and *P. armeniaca* share the same ancestor at ∼ 29.03 Mya, and the divergence of these group occurred at ∼ 30.47 Mya from the common ancestor with *P. persica* (Fig. 2A).

The *Ks* distribute analysis showed that *P. humilis* shared the same WGT-r (*Ks *=* *2.29) event with other *Prunus* species (Fig. 2B). There are no other whole genome duplication events after this event. In the *Prunus* genus, there also exist some small duplication events at recent years (*Ks *=* *0.028 – 0.122) (Fig. 2B). These duplications may be caused by tandem duplication or some interspecific hybridization.

According to the evolution relationship, *P. persica* and *P. mume* were selected to process synteny analysis with *P. humilis*. The result indicated that the genome of *P. humilis* has high similarity with other two species, the similarity rates between *P. humilis* and *P. mume* were up to 89.56% and 90.89%, respectively. And these rates between *P. humilis* and *P. persica* were 90.18% and 87.80%, respectively. Among all eight chromosomes, only chromosome 6 contained the chromosome level structure variations. And chromosome 4 has the lowest similarity rate with other two species (Fig. 2C).

After comparing all the clustered gene families in 11 plants, 2,522 expansion and 5,602 decrease families were identified (Fig. 2A). Among these expansions, only 188 gene families were significantly expanded (*P *<* *0.01) and these significant expansions included 1,042 genes (Supplementary Table S8). Among these genes, we find 17 WAX-related genes which maybe contribute to the foliar wax of *P. humilis*, and foliar wax can help plant to reduce water loss. Beside these WAX-related genes, we also identified 14 late embryogenesis abundant proteins (LEA) that are part of the expansion genes (Supplementary Table S8). Previous studies indicate *LEA* genes were highly related to the cold and drought resistance of plants. The analysis showed that only 644 families with 852 genes were specific in the *P. humilis* genome (Fig. 2D), and among these genes, two *LEA* genes were also identified and other genes such as *PIN*, *ERF/AP2* and *TCP* genes were also identified in the specific genes. So, the expansion and gain of *WAX*, *LEA* and other related genes maybe contributed to the high drought tolerance of *P. humilis.*

### 3.5. Analysis of LEA family expansion in *P. humilis*

**Figure 3:**
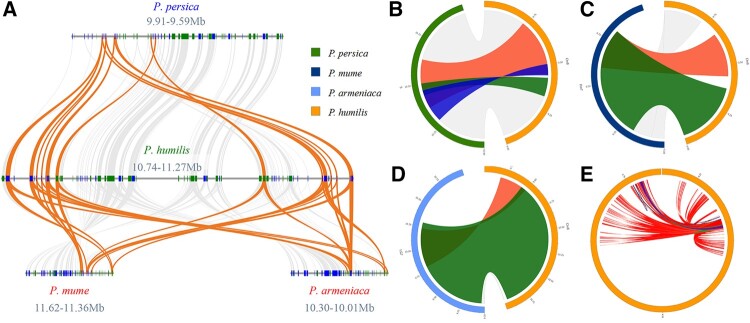
The mechanism of LEA families expansion in *P. humilis*. (A) Synteny relationship of *LEA* tandem duplication region between *P. persica, P. mume, P. armeniaca* and *P. humilis.* (B) Synteny relationship of *LEA* tandem duplication region between *P. armeniaca and P. humilis.* (C) Synteny relationship of *LEA* tandem duplication region between *P. mume* and *P. humilis.* (D) Synteny relationship of *LEA* tandem duplication region between *P. persica* and *P. humilis.* (E) LTRs in the LEA tandem duplication region of *P. humilis*.

Ks analysis of these tandem duplicated genes indicated that most of these duplications occurred in most recent years (Supplementary Fig. S7). One or several tandem duplications may contribute to the expansion of these *LEAs*. After compared with *P. mume*, *P. armeniaca*, *P. persica* and *P. humilis*, we found that a segment duplication which contained 143 LTRs contributed to this expansion (Fig. 3A). Part of these LTRs was shared by these four species, and the final segment duplication, which occurred only in *P. humilis*, doubled these expansions (Fig. 3B–E).

### 3.6. Identification and analysis of drought tolerance-related genes

**Figure 4:**
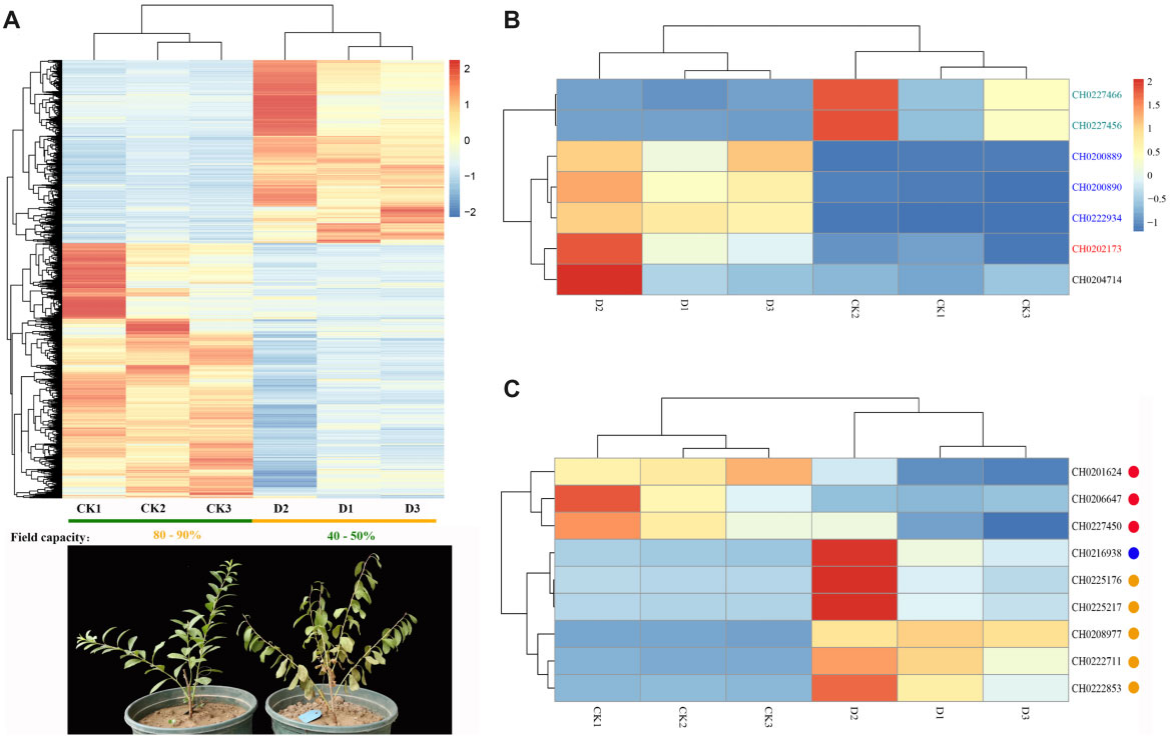
Differentially expressed genes (DEGs) of *P. humilis* under drought stress. (A) Expression profiles of DEGs under drought stress. (B) Expression of desiccation-responsive genes in *P. humilis*. CH0227466 and CH0227456 are *MYC2* genes. CH0200889, CH0200890 and CH0222934 are aldehyde dehydrogenase genes. CH0202173 is *DHN* gene. (C) Expression of drought tolerance-related genes in *P. humilis*.

RNA-seq of drought-treated and well-watered leaves was used to discover the candidate genes that are related to drought tolerance of *P. humilis*. After treatment, 5,819 genes were expressed significantly different from control, and 3,387 of these genes were up-regulated and 2,432 were down-regulated (Fig. 4A).

The GO enrichment of these DEGs indicated that most of these genes are involved in photosynthesis (GO:0015979, GO:0019684, GO:0009773, GO:0009767, GO:0009765 and GO:0009416), response to karrikin (GO:0080167) and desiccation (GO:0009269) (Supplementary Table S9). In response to desiccation, all seven genes were expressed differently, including three aldehyde dehydrogenase genes, two *MYC2* genes and a dehydrin (*DHN*) gene, which was a member of the LEA family (Fig. 4B). These results indicated that photosynthesis played an important role in drought tolerance of *P. humilis.* Photosynthesis was influenced by the drought and this influence can decrease the consumption of water and enhance the drought tolerance of plant. Among the DEGs, 19 *LEA* genes that are related to the drought tolerance were also identified. Pathway analysis of the DEGs showed that nine genes in the drought response pathway were expressed differently, including PYR/PYL (three members), PP2C (five members) and MAPKKK (one member) (Fig. 4C).

### 3.7. Population diversity analysis of *P. humilis*

**Figure 5:**
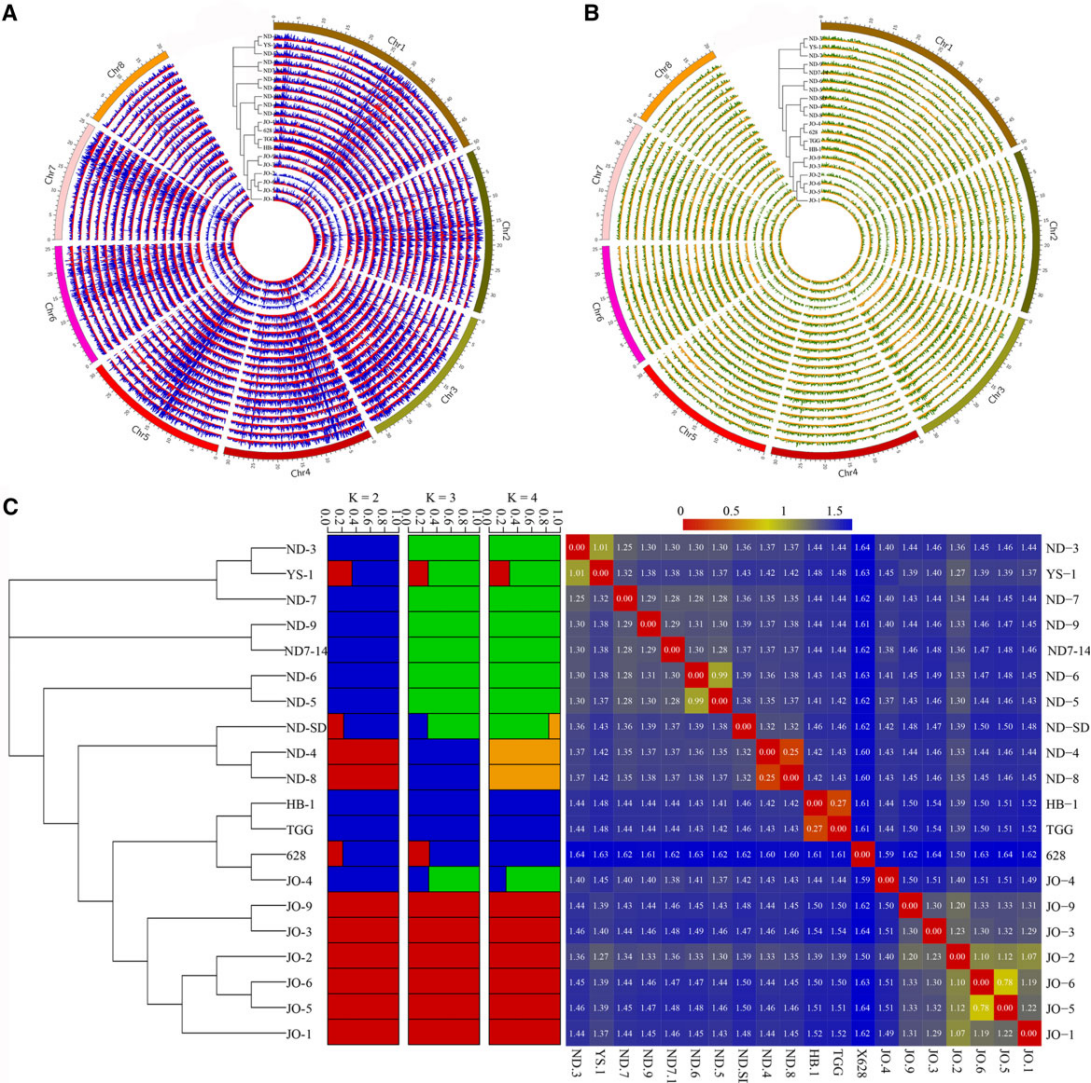
Population diversity analysis of *P. humilis*. (A) SNPs among 20 accessions of *P. humilis.* (B) Indels among 20 accessions of *P. humilis.* (C) The phylogenetic structure and DNA mutation rate among 20 accessions of *P. humilis*. The population structures were constructed with the k = 2, 3, 4. The DNA mutation rates were calculated by the SNPs and indels between two accessions. Red colour means lower mutation rate or closer relationship, and blue colour means higher difference and more distant relationship.

A population contains 20 accessions that were collected from different geographical regions in northern China were used to analyse the genetic diversity of *P. humilis.* In total, 9.40 Mb variations were obtained, including 8.48 Mb SNPs and 0.93 Mb Indels (Fig. 5A and B). Finally, 4.91 Mb high quality variations were retained after quality filter.

We found that the genetic diversity of *P. humilis* population were very low, only 1.40% base pairs were different with each other, and most of them were from 1.3% to 1.7% (Fig. 5C). The phylogenetic and population structure analysis also indicated that the population of *P. humilis* only contains two sub-populations, this is also accordance with its geographical distribution. There only exists small difference between the two groups, and accession 628 is in a special position, it has higher variation rate with other members (Fig. 5C).

## 4. Discussion

### 4.1. The genome of *P. humilis* contributes to the gene function and evolution study of *Prunus*

This study provides a high-quality chromosome-level genome sequence of *P. humilis* with the total size of 254.38 Mb and 28,301 protein-coding genes. Compared with other plants of the *Prunus* family, *P. humilis* is a bush and has a shorter juvenile phase, biennial plants can bear fruit. Meanwhile, an efficient, reliable and optimized genetic transformation system of *P. humilis* has been built.^42^ With the high similarity with other species in *Prunus*, smaller genome size with less redundant gene, shorter juvenile phase and efficient, reliable transformation system, *P. humilis* can be used as a model plant for the study of the gene function in fruit trees of *Prunus* or even the Rosaceae.

The comparison and evolution analysis among 10 Rosaceae species indicated that *P. humilis* has a close genetic relationship with *P. mume* and *P. armeniaca*, but a relatively distant relationship with *C. pseudocerasus*. Meanwhile, Synteny analysis of *P. persica*, *P. mume* and *P. humilis* indicated that the genomes of these species are highly conserved. *P. humilis* is highly chromosomal identical to *P. mume* and *P. armeniaca*, but the phenotypic differences are great among them. For example, *P. mume* and *P. armeniaca* belong to arbour, but *P. humilis* is a shrub. Therefore, the genome sequence of *P. humilis* is significant for studying on the evolution and environmental adaptation of Rosaceae.

### 4.2. LTR-derived tandem and segments duplication of *LEA* genes plays an important role in high drought resistance of *P. humilis*

As a small shrub, *P. humilis* is highly stress-resistant, especially drought-resistant.^2^ In this study, we found that LEA families in *P. humilis* were expanded, this expansion was caused by a tandem repeat event. This tandem repeat events were driven by huge number of LTRs. Besides these tandem repeat, we also identified two *LEA* genes that are specific to *P. humilis*. The transcriptome analysis also showed that *DHN* genes, a sub-family of LEA, played a key role in the high drought tolerance of *P. humilis*. Previous studies also confirmed that LEA related to the cold and drought tolerance in plants.^43–48^ So, we believe the expansion of LEA maybe an important factor that contributes to the high stress tolerance of *P. humilis*.

Our study found that the *LEA* genes in genome of *P. humilis* were expanded because of the tandem repeats contributed by LTRs, and thereby we can assume that LTRs lead to the expansion of *LEA* genes, which might contribute to the high drought tolerance of *P. humilis*.

LTRs were widely existed in the plant genomes, and usually play an important role in plant development or environment adaption.^49^^,^^50^ According to the syntenic analysis, we found that compared to its relatives, there existed a specific tandem duplication cluster of LEA in the genome of *P. humilis.* This duplication was not a single event, it is mainly caused by a duplication of a cluster contained hundreds of LTRs. And the *Ks* analysis of these tandem genes indicated that most of these tandem or segments duplications occurred in the near past (most of the gene duplication were occurred with *Ks* < 0.05). These duplications led to member increase of LEAs and could contribute to the high tolerance of cold and drought. This expansion was also find in other plant genomes.^14^^,^^51^ So, LTRs maybe one of the drive factor of the environment adaption in *P. humilis.*

### 4.3. Studies on germplasm provided evidence for the origin and variety improvement of *P. humilis*

Origin and spread is always important for plant diversity and evolution analysis. As a special fruit tree native to China, *P. humilis* has a limited native distribution. They are only naturally distributed in the northern China. Population diversity analysis of *P. humilis* demonstrated that there was little difference and no obvious cluster of differentiation among *P. humilis* populations. We collected these accessions over a larger region in North China that were significant difference of the climate and geographic conditions among these accessions. These low genetic diversity may suggest that the spread of these accessions occurred in the near past or sufficient gene exchange has occurred. According to the low diversity of these accessions, it was hard to locate the origin area of *C. humilis*, but the present evidence supported that *P. humilis* originated in northern China and distributed in this region only.

With the emerging industry of fruit trees, most *P. humilis* varieties were mainly collected directly from the field and no more domestication occurred. So, the domestication and modern breeding or improvement of *P. humilis* is necessary. Population diversity indicated that although high homogeneity existed among the present population, some germplasm such as 628 has a higher genetic difference from others. So due to limited genetic diversity now, the collection of more wild accessions is necessary for genetic diversity study and germplasm improvement. In addition, hybridization between high heterogeneity accessions could also contribute to the germplasm improvement, and this may be a good choice for the breeding of *P. humilis*. On the other hand, human selection which could significantly accelerate the breeding programme also should be processed, these selection could improve the varieties according to the will of human beings. In a nutshell, as a new emerged fruit, more useful strategies such as germplasm collection, hybridization and human selection should be adopted to breed high-quality cultivars.

## Supplementary Material

dsac021_Supplementary_Data
