# Correction to: The genome of *Prunus humilis* provides new insights to drought adaption and population diversity

**DOI:** 10.1093/dnares/dsac038

**Published:** 2022-12-23

This is a correction to: Yi Wang, Jun Xie, Hongna Zhang, Weidong Li, Zhanjun Wang, Huayang Li, Qian Tong, Gaixia Qiao, Yujuan Liu, Ying Tian, Yongzan Wei, Ping Li, Rong Wang, Weiping Chen, Zhengchang Liang, Meilong Xu, The genome of Prunus humilis provides new insights to drought adaption and population diversity, DNA Research, Volume 29, Issue 4, August 2022, dsac021, https://doi.org/10.1093/dnares/dsac021. In the originally published version of this manuscript, there were errors in the order of the affiliations. These errors have been corrected.

