# Supplementary material for: The genome of Prunus humilis provides new insights to drought adaption and population diversity
Source: DNA Res. 2022 Jun 25;29(4):dsac021. doi: 10.1093/dnares/dsac021 (PMC9278622; doi:10.1093/dnares/dsac021)
Supplement: dsac021_Supplementary_Data [file dsac021_supplementary_data.zip › S Table.docx]

**Table S1 Genome survey of five** ***C. humilis* varieties**

| **accessions** | ***k-mer*** | | | **Genome Size** |
| --- | --- | --- | --- | --- |
|  | **17** | **19** | **21** |  |
| JO-1 | 0.700% | 0.717% | 0.717% | 252,663,766 |
| JO-2 | 0.681% | 0.698% | 0.697% | 247,179,078 |
| ND-4 | 0.700% | 0.715% | 0.713% | 242,767,782 |
| ND-5 | 0.702% | 0.719% | 0.735% | 242,494,434 |
| ND-6 | 0.742% | 0.759% | 0.744% | 247,165,935 |

**Table S2 Raw data used to constructed the genome of *C. humilis***

| **sequencing platform** | **enzyme** | **data size** |
| --- | --- | --- |
| Illumina | HindIII | 23.34Gb |
| Pacbio |  | 27.49Gb |
| Hi-C |  | 126.64Gb |

**Table S3 Detail information of the raw data, corrected and trimmed Pacbio reads, and contigs**

|  | Raw Data | Corrected Data | Trimmed Data | Contigs |
| --- | --- | --- | --- | --- |
| Total Length | 27,493,699,923 | 9,312,035,066 | 7,477,613,616 | 253,654,080 |
| Sequence Number | 2,334,777 | 430,147 | 428,930 | 1,532 |
| Longest sequence | 207,969 | 134,707 | 109,677 | 2,689,212 |
| Shortest sequence | 50 | 6 | 1,000 | 16,717 |
| N10 | 29,086 | 33,746 | 26,854 | 1,333,893 |
| N20 | 23,609 | 28,776 | 23,488 | 901,625 |
| N30 | 20,189 | 25,907 | 21,487 | 692,424 |
| N40 | 17,262 | 23,834 | 20,002 | 544,055 |
| N50 | 14,941 | 22,191 | 18,787 | 381,959 |
| N60 | 13,150 | 20,765 | 17,758 | 277,937 |
| N70 | 11,696 | 19,455 | 16,862 | 172,895 |
| N80 | 10,297 | 18,202 | 14,741 | 90,736 |
| N90 | 7,858 | 17,050 | 11,915 | 55,240 |

**Table S4 BUSCO result of *C. humilis* genome**

| BUSCO was run in mode: genome | |
| --- | --- |
| C:96.3%[S:91.0%,D:5.3%],F:1.0%,M:2.7%,n:2121 | |
| Gene number | Type |
| 2043 | Complete BUSCOs (C) |
| 1931 | Complete and single-copy BUSCOs (S) |
| 112 | Complete and duplicated BUSCOs (D) |
| 22 | Fragmented BUSCOs (F) |
| 56 | Missing BUSCOs (M) |
| 2121 | Total BUSCO groups searched |

**Table S5 RNA-seq information of fruit, stem and leaf**

| tissue | input | maped | mapping rate |
| --- | --- | --- | --- |
| fruit | 46145278 | 43094858 | 93.4 |
| stem | 42526782 | 40407529 | 95 |
| leaf | 41956824 | 39901892 | 95.1 |

**Table S6 The repetitive sequences information of the whole genome**

| type | number of elements | length(bp) | percentage of sequence (%) |
| --- | --- | --- | --- |
| SINEs | 9501 | 1079910 | 0.42 |
| LINEs | 8730 | 3211242 | 1.26 |
| LTR elements | 103279 | 48270572 | 18.98 |
| DNA elements | 93393 | 30261512 | 11.90 |
| Unclassified | 78530 | 22967523 | 9.03 |

**Table S7 The LTR information of the whole genome**

| LTR types | Length (bp) | Number | Percentage |
| --- | --- | --- | --- |
| LTR/Copia | 5843924 | 13217 | 0.10663 |
| LTR/Gypsy | 19707344 | 23545 | 0.18995 |
| LTR/unknown | 22968169 | 87194 | 0.70343 |
| Total | 48519437 | 123956 | 1 |

**Table S8 The information of significant expansion genes**

| Gene ID | Gene annotation |
| --- | --- |
| CH0203609 | Inherit from KOG: Retrotransposon protein |
| CH0203691 | Inherit from KOG: Retrotransposon protein |
| CH0203738 | Inherit from KOG: Retrotransposon protein |
| CH0203914 | Inherit from KOG: Retrotransposon protein |
| CH0204067 | Inherit from KOG: Retrotransposon protein |
| CH0204077 | Inherit from KOG: Retrotransposon protein |
| CH0204078 | Inherit from KOG: Retrotransposon protein |
| CH0204877 | Inherit from KOG: Retrotransposon protein |
| CH0207797 | Inherit from KOG: Retrotransposon protein |
| CH0207799 | Inherit from KOG: Retrotransposon protein |
| CH0207870 | Inherit from KOG: Retrotransposon protein |
| CH0207875 | Inherit from KOG: Retrotransposon protein |
| CH0207889 | Inherit from KOG: Retrotransposon protein |
| CH0208016 | Inherit from KOG: Retrotransposon protein |
| CH0208020 | Inherit from KOG: Retrotransposon protein |
| CH0211956 | Inherit from KOG: Retrotransposon protein |
| CH0212002 | Inherit from KOG: Retrotransposon protein |
| CH0212171 | Inherit from KOG: Retrotransposon protein |
| CH0212271 | Inherit from KOG: Retrotransposon protein |
| CH0212277 | Inherit from KOG: Retrotransposon protein |
| CH0212289 | Inherit from KOG: Retrotransposon protein |
| CH0212544 | Inherit from KOG: Retrotransposon protein |
| CH0212671 | Inherit from KOG: Retrotransposon protein |
| CH0213391 | Inherit from KOG: Retrotransposon protein |
| CH0213569 | Inherit from KOG: Retrotransposon protein |
| CH0217683 | Inherit from KOG: Retrotransposon protein |
| CH0217692 | Inherit from KOG: Retrotransposon protein |
| CH0217755 | Inherit from KOG: Retrotransposon protein |
| CH0217844 | Inherit from KOG: Retrotransposon protein |
| CH0221474 | Inherit from KOG: Retrotransposon protein |
| CH0221866 | Inherit from KOG: Retrotransposon protein |
| CH0224621 | Inherit from KOG: Retrotransposon protein |
| CH0224623 | Inherit from KOG: Retrotransposon protein |
| CH0224752 | Inherit from KOG: Retrotransposon protein |
| CH0224780 | Inherit from KOG: Retrotransposon protein |
| CH0224808 | Inherit from KOG: Retrotransposon protein |
| CH0225001 | Inherit from KOG: Retrotransposon protein |
| CH0217685 |  |
| CH0226586 |  |
| CH0217686 |  |
| CH0224638 |  |
| CH0220842 |  |
| CH0200703 | Sugar transport protein |
| CH0210606 | hexose carrier protein |
| CH0210608 | hexose carrier protein |
| CH0210610 | hexose carrier protein |
| CH0210611 | hexose carrier protein |
| CH0215630 | sugar transporter 1 |
| CH0204231 | sugar transport protein |
| CH0204232 | sugar transport protein |
| CH0204235 | sugar transport protein |
| CH0204236 | sugar transport protein |
| CH0204238 | sugar transport protein |
| CH0204239 | sugar transport protein |
| CH0204240 | sugar transport protein |
| CH0204241 | sugar transport protein |
| CH0204242 | Sugar (and other) transporter |
| CH0204259 | sugar transport protein |
| CH0204260 | sugar transport protein |
| CH0204261 | sugar transport protein |
| CH0204262 | sugar transport protein |
| CH0205123 | Sugar carrier protein |
| CH0222421 | sugar transport protein |
| CH0222692 | Sugar transport protein |
| CH0205122 | Sugar carrier protein |
| CH0226887 | Sugar (and other) transporter |
| CH0226889 | Sugar (and other) transporter |
| CH0204237 | sugar transport protein |
| CH0204263 | sugar transport protein |
| CH0205917 | Sugar carrier protein |
| CH0226966 | Sugar (and other) transporter |
| CH0210607 | hexose carrier protein |
| CH0201792 | L-type lectin-domain containing receptor kinase IX.1-like |
| CH0220832 | L-type lectin-domain containing receptor kinase IX.1-like |
| CH0220845 | L-type lectin-domain containing receptor kinase IX.1-like |
| CH0207382 | L-type lectin-domain containing receptor kinase IX.1-like |
| CH0207384 | L-type lectin-domain containing receptor kinase IX.1-like |
| CH0214320 | L-type lectin-domain containing receptor kinase IX.1-like |
| CH0214326 | L-type lectin-domain containing receptor kinase IX.1-like |
| CH0214297 | L-type lectin-domain containing receptor kinase IX.1-like |
| CH0214299 | L-type lectin-domain containing receptor kinase IX.1-like |
| CH0214313 | L-type lectin-domain containing receptor kinase IX.1-like |
| CH0214314 | L-type lectin-domain containing receptor kinase IX.1-like |
| CH0214319 | L-type lectin-domain containing receptor kinase IX.1-like |
| CH0213675 | L-type lectin-domain containing receptor kinase IX.1-like |
| CH0213677 | L-type lectin-domain containing receptor kinase IX.1-like |
| CH0214295 | L-type lectin-domain containing receptor kinase IX.1-like |
| CH0214324 | L-type lectin-domain containing receptor kinase IX.1-like |
| CH0214296 | L-type lectin-domain containing receptor kinase IX.1-like |
| CH0214300 | L-type lectin-domain containing receptor kinase IX.1-like |
| CH0214308 | L-type lectin-domain containing receptor kinase IX.1-like |
| CH0214316 | L-type lectin-domain containing receptor kinase IX.1-like |
| CH0214302 | L-type lectin-domain containing receptor kinase IX.1-like |
| CH0214318 | L-type lectin-domain containing receptor kinase IX.1-like |
| CH0214303 | L-type lectin-domain containing receptor kinase IX.1-like |
| CH0214305 | L-type lectin-domain containing receptor kinase IX.1-like |
| CH0214309 | L-type lectin-domain containing receptor kinase IX.1-like |
| CH0214304 | L-type lectin-domain containing receptor kinase IX.1-like |
| CH0214322 | L-type lectin-domain containing receptor kinase IX.1-like |
| CH0228188 | L-type lectin-domain containing receptor kinase IX.1-like |
| CH0201793 | L-type lectin-domain containing receptor kinase IX.1-like |
| CH0214294 | L-type lectin-domain containing receptor kinase IX.1-like |
| CH0200089 | exostosin family |
| CH0204119 | exostosin family |
| CH0204126 | exostosin family |
| CH0208937 | exostosin family |
| CH0209650 | exostosin family |
| CH0200090 | Elongation factor |
| CH0200091 | Elongation factor |
| CH0200394 | Elongation factor |
| CH0204100 | exostosin family |
| CH0204116 | exostosin family |
| CH0204134 | exostosin family |
| CH0204135 | exostosin family |
| CH0204136 | exostosin family |
| CH0204138 | exostosin family |
| CH0204139 | exostosin family |
| CH0224707 | exostosin family |
| CH0204120 | exostosin family |
| CH0204123 | exostosin family |
| CH0219913 | exostosin family |
| CH0201392 | Patatin group |
| CH0201394 | Patatin group |
| CH0201395 | Patatin group |
| CH0201396 | Patatin group |
| CH0201397 | Patatin group |
| CH0201398 | Patatin group |
| CH0201402 | Patatin group |
| CH0227865 | Patatin group |
| CH0227867 | Patatin group |
| CH0227868 | Patatin group |
| CH0227869 | Patatin group |
| CH0227870 | Patatin group |
| CH0227871 | Patatin group |
| CH0227873 | Patatin group |
| CH0227878 | Patatin group |
| CH0201393 | Patatin group |
| CH0227866 | Patatin group |
| CH0201399 | Patatin group |
| CH0201401 | Patatin group |
| CH0227872 | Patatin group |
| CH0227879 | Patatin group |
| CH0201403 | Patatin group |
| CH0227876 | Patatin group |
| CH0223577 | Patatin group |
| CH0203256 | Mediator complex subunit 28 |
| CH0203257 | Mediator complex subunit 28 |
| CH0226154 | Mediator complex subunit 28 |
| CH0205290 | Mediator complex subunit 28 |
| CH0205291 | Mediator complex subunit 28 |
| CH0205292 | Mediator complex subunit 28 |
| CH0205294 | Mediator complex subunit 28 |
| CH0205297 | Mediator complex subunit 28 |
| CH0205513 | Mediator complex subunit 28 |
| CH0205518 | Mediator complex subunit 28 |
| CH0205520 | Mediator complex subunit 28 |
| CH0205521 | Mediator complex subunit 28 |
| CH0205522 | Mediator complex subunit 28 |
| CH0205524 | Mediator complex subunit 28 |
| CH0205295 | Mediator complex subunit 28 |
| CH0205296 | Mediator complex subunit 28 |
| CH0205515 | Mediator complex subunit 28 |
| CH0205514 | Mediator complex subunit 28 |
| CH0226157 | Mediator complex subunit 28 |
| CH0228209 | Mediator complex subunit 28 |
| CH0218092 | Disease resistance protein |
| CH0218307 | Disease resistance protein |
| CH0218328 | Disease resistance protein |
| CH0218329 | disease resistance protein |
| CH0218628 | Disease resistance protein |
| CH0228219 | Disease resistance protein |
| CH0218267 | disease resistance protein |
| CH0218270 | disease resistance protein |
| CH0218273 | Disease resistance protein |
| CH0218276 | Disease resistance protein |
| CH0218278 | disease resistance protein |
| CH0218279 | Disease resistance protein |
| CH0218302 | Disease resistance protein |
| CH0218306 | disease resistance protein |
| CH0218309 | disease resistance protein |
| CH0218332 | disease resistance protein |
| CH0218333 | Disease resistance protein |
| CH0218334 | Disease resistance protein |
| CH0219278 | Disease resistance protein |
| CH0219281 | Disease resistance protein |
| CH0228218 | disease resistance protein |
| CH0208337 | Pfam:MuDR |
| CH0208358 | Pfam:MuDR |
| CH0208342 | Pfam:MuDR |
| CH0208354 | Pfam:MuDR |
| CH0217515 | Pfam:MuDR |
| CH0224373 | Pfam:MuDR |
| CH0213557 | Pfam:MuDR |
| CH0217467 | Pfam:MuDR |
| CH0218191 | Pfam:MuDR |
| CH0217518 | Pfam:MuDR |
| CH0225193 | Pfam:MuDR |
| CH0217797 | Pfam:MuDR |
| CH0217187 | Pfam:MuDR |
| CH0223079 | ankyrin repeat-containing protein |
| CH0223081 | ankyrin repeat-containing protein |
| CH0223082 | Ankyrin repeat-containing protein |
| CH0223083 | ankyrin repeat-containing protein |
| CH0223084 | ankyrin repeat-containing protein |
| CH0223112 | Ankyrin repeat-containing protein |
| CH0223114 | ankyrin repeat-containing protein |
| CH0223115 | ankyrin repeat-containing protein |
| CH0214061 | ANK |
| CH0221765 | benzyl alcohol O-benzoyltransferase-like |
| CH0221790 | benzyl alcohol O-benzoyltransferase-like |
| CH0227626 | benzyl alcohol O-benzoyltransferase-like |
| CH0227627 | benzyl alcohol O-benzoyltransferase-like |
| CH0227629 | benzyl alcohol O-benzoyltransferase-like |
| CH0227636 | benzyl alcohol O-benzoyltransferase-like |
| CH0227664 | benzyl alcohol O-benzoyltransferase-like |
| CH0227665 | benzyl alcohol O-benzoyltransferase-like |
| CH0227632 | benzyl alcohol O-benzoyltransferase-like |
| CH0227635 | benzyl alcohol O-benzoyltransferase-like |
| CH0227637 | benzyl alcohol O-benzoyltransferase-like |
| CH0227659 | benzyl alcohol O-benzoyltransferase-like |
| CH0223288 | mitochondrial chaperone |
| CH0223347 | mitochondrial chaperone |
| CH0223348 | mitochondrial chaperone |
| CH0223356 | mitochondrial chaperone |
| CH0223357 | mitochondrial chaperone |
| CH0223360 | mitochondrial chaperone |
| CH0223346 | mitochondrial chaperone |
| CH0223355 | mitochondrial chaperone |
| CH0218391 | NA |
| CH0218406 | coatomer protein complex, subunit beta 2 (beta prime) |
| CH0218401 | coatomer protein complex, subunit beta 2 (beta prime) |
| CH0218426 | coatomer protein complex, subunit beta 2 (beta prime) |
| CH0218432 | coatomer protein complex, subunit beta 2 (beta prime) |
| CH0222647 | coatomer protein complex, subunit beta 2 (beta prime) |
| CH0218402 | coatomer protein complex, subunit beta 2 (beta prime) |
| CH0218427 | coatomer protein complex, subunit beta 2 (beta prime) |
| CH0218428 | coatomer protein complex, subunit beta 2 (beta prime) |
| CH0218429 | coatomer protein complex, subunit beta 2 (beta prime) |
| CH0218433 | coatomer protein complex, subunit beta 2 (beta prime) |
| CH0218434 | coatomer protein complex, subunit beta 2 (beta prime) |
| CH0218424 | coatomer protein complex, subunit beta 2 (beta prime) |
| CH0202737 | Ubox domain-containing protein |
| CH0202759 | Ubox domain-containing protein |
| CH0202761 | Ubox domain-containing protein |
| CH0202740 | DNA-dependent RNA polymerase catalyzes the transcription of DNA into RNA using the four ribonucleoside triphosphates as substrates (By similarity) |
| CH0202757 | DNA-dependent RNA polymerase catalyzes the transcription of DNA into RNA using the four ribonucleoside triphosphates as substrates (By similarity) |
| CH0216632 | DNA-dependent RNA polymerase catalyzes the transcription of DNA into RNA using the four ribonucleoside triphosphates as substrates (By similarity) |
| CH0222293 | DNA-dependent RNA polymerase catalyzes the transcription of DNA into RNA using the four ribonucleoside triphosphates as substrates (By similarity) |
| CH0228102 | DNA-dependent RNA polymerase catalyzes the transcription of DNA into RNA using the four ribonucleoside triphosphates as substrates (By similarity) |
| CH0228107 | DNA-dependent RNA polymerase catalyzes the transcription of DNA into RNA using the four ribonucleoside triphosphates as substrates (By similarity) |
| CH0202736 | Ubox domain-containing protein |
| CH0208249 | fatty acyl-CoA reductase |
| CH0208255 | fatty acyl-CoA reductase |
| CH0208312 | fatty acyl-CoA reductase |
| CH0208318 | fatty acyl-CoA reductase |
| CH0208322 | fatty acyl-CoA reductase |
| CH0208323 | fatty acyl-CoA reductase |
| CH0208325 | fatty acyl-CoA reductase |
| CH0208326 | fatty acyl-CoA reductase |
| CH0208368 | fatty acyl-CoA reductase |
| CH0208369 | fatty acyl-CoA reductase |
| CH0208317 | fatty acyl-CoA reductase |
| CH0223624 | transferase, family |
| CH0223627 | transferase, family |
| CH0223630 | transferase, family |
| CH0223646 | transferase, family |
| CH0223647 | transferase, family |
| CH0223648 | transferase, family |
| CH0223649 | transferase, family |
| CH0223650 | transferase, family |
| CH0223645 | transferase, family |
| CH0223631 | transferase, family |
| CH0223634 | transferase, family |
| CH0219130 | Acyl-CoA synthetase long-chain family member |
| CH0219132 | Acyl-CoA synthetase long-chain family member |
| CH0220908 | Acyl-CoA synthetase long-chain family member |
| CH0220937 | Acyl-CoA synthetase long-chain family member |
| CH0225178 | Acyl-CoA synthetase long-chain family member |
| CH0225215 | Acyl-CoA synthetase long-chain family member |
| CH0221283 | UPF0481 protein |
| CH0224554 | UPF0481 protein |
| CH0224572 | UPF0481 protein |
| CH0224552 | UPF0481 protein |
| CH0224570 | UPF0481 protein |
| CH0224553 | UPF0481 protein |
| CH0224556 | UPF0481 protein |
| CH0224573 | UPF0481 protein |
| CH0224574 | UPF0481 protein |
| CH0224575 | UPF0481 protein |
| CH0224580 | UPF0481 protein |
| CH0224555 | UPF0481 protein |
| CH0224576 | UPF0481 protein |
| CH0224579 | UPF0481 protein |
| CH0210093 | Dehydrogenase |
| CH0220585 | Dehydrogenase |
| CH0220586 | Dehydrogenase |
| CH0221812 | Dehydrogenase |
| CH0221813 | Dehydrogenase |
| CH0221814 | Dehydrogenase |
| CH0211724 | WAX2-like |
| CH0211726 | WAX2-like |
| CH0211727 | WAX2-like |
| CH0211732 | WAX2-like |
| CH0211750 | WAX2-like |
| CH0211756 | WAX2-like |
| CH0211755 | WAX2-like |
| CH0201759 | aspartic proteinase |
| CH0201761 | aspartic proteinase |
| CH0201763 | aspartic proteinase |
| CH0201764 | aspartic proteinase |
| CH0201765 | aspartic proteinase |
| CH0201766 | aspartic proteinase |
| CH0201767 | aspartic proteinase |
| CH0201770 | aspartic proteinase |
| CH0201771 | aspartic proteinase |
| CH0201772 | aspartic proteinase |
| CH0201773 | aspartic proteinase |
| CH0201774 | aspartic proteinase |
| CH0204934 | aspartic proteinase |
| CH0201760 | aspartic proteinase |
| CH0204963 | aspartic proteinase |
| CH0201858 | Molybdenum cofactor sulfurase |
| CH0223475 | Molybdenum cofactor sulfurase |
| CH0223476 | Molybdenum cofactor sulfurase |
| CH0223510 | Molybdenum cofactor sulfurase |
| CH0223512 | Molybdenum cofactor sulfurase |
| CH0223511 | Molybdenum cofactor sulfurase |
| CH0201860 | B3 domain-containing protein |
| CH0204876 | Conserved gene of |
| CH0204930 | Conserved gene of |
| CH0210304 | Conserved gene of |
| CH0213494 | Conserved gene of |
| CH0215147 | Conserved gene of |
| CH0217804 | Conserved gene of |
| CH0220838 | gibberellin 20 oxidase |
| CH0220839 | gibberellin 20 oxidase |
| CH0220840 | gibberellin 20 oxidase |
| CH0220868 | gibberellin 20 oxidase |
| CH0220869 | gibberellin 20 oxidase |
| CH0220871 | gibberellin 20 oxidase |
| CH0220872 | gibberellin 20 oxidase |
| CH0220870 | gibberellin 20 oxidase |
| CH0220873 | gibberellin 20 oxidase |
| CH0218550 | ankyrin repeat-containing protein |
| CH0218552 | ankyrin repeat-containing protein |
| CH0218557 | Ankyrin repeat-containing protein |
| CH0218558 | Ankyrin repeat-containing protein |
| CH0218561 | ankyrin repeat-containing protein |
| CH0218555 | Ankyrin repeat-containing protein |
| CH0218559 | Ankyrin repeat-containing protein |
| CH0218563 | ankyrin repeat-containing protein |
| CH0218562 | ankyrin repeat-containing protein |
| CH0204257 | Transcription regulatory protein |
| CH0204399 | SWI SNF related, matrix associated, actin dependent regulator of chromatin, subfamily a, member |
| CH0204258 | chromosome 9 open reading frame 114 |
| CH0204264 | chromosome 9 open reading frame 114 |
| CH0204269 | chromosome 9 open reading frame 114 |
| CH0204400 | SWI SNF related, matrix associated, actin dependent regulator of chromatin, subfamily a, member |
| CH0222109 | tryptophan aminotransferase-related protein 4-like |
| CH0222113 | tryptophan aminotransferase-related protein 4-like |
| CH0222110 | tryptophan aminotransferase-related protein 4-like |
| CH0222114 | tryptophan aminotransferase-related protein 4-like |
| CH0222115 | tryptophan aminotransferase-related protein 4-like |
| CH0222121 | tryptophan aminotransferase-related protein 4-like |
| CH0200085 | Leucyl-trna synthetase |
| CH0212810 | Leucyl-trna synthetase |
| CH0212811 | Leucyl-trna synthetase |
| CH0213024 | Leucyl-trna synthetase |
| CH0213238 | Leucyl-trna synthetase |
| CH0213025 | Leucyl-trna synthetase |
| CH0213237 | Leucyl-trna synthetase |
| CH0220688 | Pfam:UPF0089 |
| CH0220699 | Pfam:UPF0089 |
| CH0220705 | Pfam:UPF0089 |
| CH0220706 | Pfam:UPF0089 |
| CH0220708 | Pfam:UPF0089 |
| CH0220733 | Pfam:UPF0089 |
| CH0220734 | Pfam:UPF0089 |
| CH0220707 | Pfam:UPF0089 |
| CH0220732 | Pfam:UPF0089 |
| CH0220709 | Pfam:UPF0089 |
| CH0220731 | Pfam:UPF0089 |
| CH0203889 | UDP-glycosyltransferase 92A1-like |
| CH0203891 | UDP-glycosyltransferase 92A1-like |
| CH0203893 | UDP-glycosyltransferase 92A1-like |
| CH0203895 | UDP-glycosyltransferase 92A1-like |
| CH0203935 | UDP-glycosyltransferase 92A1-like |
| CH0203946 | UDP-glycosyltransferase 92A1-like |
| CH0203939 | UDP-glycosyltransferase 92A1-like |
| CH0203944 | UDP-glycosyltransferase 92A1-like |
| CH0200857 | GDSL-like Lipase/Acylhydrolase |
| CH0200859 | GDSL-like Lipase/Acylhydrolase |
| CH0200861 | GDSL-like Lipase/Acylhydrolase |
| CH0200862 | GDSL-like Lipase/Acylhydrolase |
| CH0219416 | amidase C869.01-like |
| CH0219417 | amidase C869.01-like |
| CH0220502 | amidase C869.01-like |
| CH0220503 | amidase C869.01-like |
| CH0220504 | amidase C869.01-like |
| CH0219421 | amidase C869.01-like |
| CH0220505 | amidase C869.01-like |
| CH0220506 | amidase C869.01-like |
| CH0226602 | Late embryogenesis abundant protein |
| CH0226659 | Late embryogenesis abundant protein |
| CH0226661 | Late embryogenesis abundant protein |
| CH0226669 | Late embryogenesis abundant protein |
| CH0226608 | Late embryogenesis abundant protein |
| CH0226624 | Late embryogenesis abundant protein |
| CH0226667 | Late embryogenesis abundant protein |
| CH0226603 | Late embryogenesis abundant protein |
| CH0226662 | Late embryogenesis abundant protein |
| CH0226604 | Late embryogenesis abundant protein |
| CH0226663 | Late embryogenesis abundant protein |
| CH0226605 | Late embryogenesis abundant protein |
| CH0226664 | Late embryogenesis abundant protein |
| CH0226609 | Late embryogenesis abundant protein |
| CH0218895 | hydroxycinnamoyl-Coenzyme A shikimate quinate hydroxycinnamoyltransferase-like |
| CH0218923 | hydroxycinnamoyl-Coenzyme A shikimate quinate hydroxycinnamoyltransferase-like |
| CH0228143 | hydroxycinnamoyl-Coenzyme A shikimate quinate hydroxycinnamoyltransferase-like |
| CH0228144 | hydroxycinnamoyl-Coenzyme A shikimate quinate hydroxycinnamoyltransferase-like |
| CH0228146 | hydroxycinnamoyl-Coenzyme A shikimate quinate hydroxycinnamoyltransferase-like |
| CH0203540 |  |
| CH0204889 |  |
| CH0211700 |  |
| CH0212052 |  |
| CH0214669 |  |
| CH0224608 |  |
| CH0227631 |  |
| CH0209267 |  |
| CH0209743 |  |
| CH0212352 |  |
| CH0222118 |  |
| CH0227877 | NA |
| CH0203696 | NA |
| CH0204130 | NA |
| CH0207120 | NA |
| CH0212890 | Retrotransposon protein |
| CH0213028 | NA |
| CH0213115 | NA |
| CH0213455 | NA |
| CH0219754 | NA |
| CH0221835 | NA |
| CH0224945 | NA |
| CH0225103 | NA |
| CH0226832 | NA |
| CH0228100 | NA |
| CH0203838 |  |
| CH0204552 |  |
| CH0211804 |  |
| CH0211818 |  |
| CH0212083 |  |
| CH0213691 |  |
| CH0214452 | Cell division cycle 16 homolog (S. cerevisiae) |
| CH0218248 |  |
| CH0224138 |  |
| CH0206870 | WD repeat domain 18 |
| CH0208367 |  |
| CH0211895 |  |
| CH0214470 |  |
| CH0223916 |  |
| CH0212669 | u3 small nucleolar ribonucleoprotein |
| CH0212670 | u3 small nucleolar ribonucleoprotein |
| CH0221214 | u3 small nucleolar ribonucleoprotein |
| CH0221222 | u3 small nucleolar ribonucleoprotein |
| CH0212672 | tetratricopeptide repeat protein 27 homolog |
| CH0204983 | Homogentisate phytyltransferase |
| CH0205004 | Homogentisate phytyltransferase |
| CH0204984 | lysyl-trna synthetase |
| CH0204986 | lysyl-trna synthetase |
| CH0204996 | lysyl-trna synthetase |
| CH0205005 | lysyl-trna synthetase |
| CH0210043 | NA |
| CH0226787 | protein transport protein-related |
| CH0226801 | protein transport protein-related |
| CH0226788 | protein transport protein-related |
| CH0226802 | protein transport protein-related |
| CH0202481 | Retrotransposon protein |
| CH0213345 | Retrotransposon protein |
| CH0214773 | Retrotransposon protein |
| CH0220961 | Retrotransposon protein |
| CH0221293 | Retrotransposon protein |
| CH0224453 | Retrotransposon protein |
| CH0204760 | Endonuclease/Exonuclease/phosphatase family |
| CH0214954 | Inherit from KOG: Retrotransposon protein |
| CH0224259 | Inherit from KOG: Retrotransposon protein |
| CH0217645 | Retrotransposon protein |
| CH0204572 | transposon protein |
| CH0204573 | transposon protein |
| CH0204579 | transposon protein |
| CH0209030 | transposon protein |
| CH0213825 | transposon protein |
| CH0221466 | transposon protein |
| CH0210908 | transposon protein |
| CH0213502 | transposon protein |
| CH0200354 | Regulator of chromosome condensation |
| CH0200359 | Regulator of chromosome condensation |
| CH0202675 | Regulator of chromosome condensation |
| CH0216004 | Regulator of chromosome condensation |
| CH0224318 | Regulator of chromosome condensation |
| CH0224321 | Regulator of chromosome condensation |
| CH0225212 | Regulator of chromosome condensation |
| CH0203757 | Regulator of chromosome condensation |
| CH0215757 | NA |
| CH0228158 | NA |
| CH0228163 | NA |
| CH0215759 | NA |
| CH0215776 | NA |
| CH0228160 | NA |
| CH0228161 | NA |
| CH0214665 | phosphoglycerate kinase |
| CH0214666 | phosphoglycerate kinase |
| CH0214679 | phosphoglycerate kinase |
| CH0214680 | phosphoglycerate kinase |
| CH0204162 | Inherit from KOG: Retrotransposon protein |
| CH0218066 | Retrotransposon protein |
| CH0204890 |  |
| CH0204895 | Retrotransposon protein |
| CH0212051 |  |
| CH0214668 |  |
| CH0227630 |  |
| CH0212587 | Retrotransposon protein |
| CH0214331 | UDP-glycosyltransferase |
| CH0214351 | UDP-glycosyltransferase |
| CH0214352 | UDP-glycosyltransferase |
| CH0214333 | UDP-glycosyltransferase |
| CH0214348 | UDP-glycosyltransferase |
| CH0203602 | Filamin/ABP280 repeat |
| CH0204505 | Filamin/ABP280 repeat |
| CH0204509 | Filamin/ABP280 repeat |
| CH0228235 | Filamin/ABP280 repeat |
| CH0201407 | Reverse transcriptase (RNA-dependent DNA polymerase) |
| CH0201494 | Reverse transcriptase (RNA-dependent DNA polymerase) |
| CH0208631 | Inherit from KOG: Retrotransposon protein |
| CH0211206 | Reverse transcriptase (RNA-dependent DNA polymerase) |
| CH0213815 | Retrotransposon protein |
| CH0219004 | Reverse transcriptase (RNA-dependent DNA polymerase) |
| CH0203832 | Survival motor neuron (SMN) interacting protein 1 (SIP1) |
| CH0203835 | Survival motor neuron (SMN) interacting protein 1 (SIP1) |
| CH0203837 | Survival motor neuron (SMN) interacting protein 1 (SIP1) |
| CH0203836 | Survival motor neuron (SMN) interacting protein 1 (SIP1) |
| CH0203840 | Survival motor neuron (SMN) interacting protein 1 (SIP1) |
| CH0203842 | Survival motor neuron (SMN) interacting protein 1 (SIP1) |
| CH0212532 | FAD binding domain |
| CH0212552 | FAD binding domain |
| CH0222036 | FAD binding domain |
| CH0222038 | FAD binding domain |
| CH0213082 | CACTA, En Spm sub-class |
| CH0213461 | CACTA, En Spm sub-class |
| CH0224982 | CACTA, En Spm sub-class |
| CH0213634 | CACTA, En Spm sub-class |
| CH0213722 |  |
| CH0221851 |  |
| CH0226773 | synthetase |
| CH0226774 | synthetase |
| CH0226790 | synthetase |
| CH0226792 | synthetase |
| CH0201262 | Beta-lactamase |
| CH0201264 | Beta-lactamase |
| CH0227925 | Beta-lactamase |
| CH0227927 | Beta-lactamase |
| CH0203720 | ribosomal protein, L38 |
| CH0203733 | ribosomal protein, L38 |
| CH0203736 | ribosomal protein, L38 |
| CH0224681 | ribosomal protein, L38 |
| CH0203793 | ribosomal protein, L38 |
| CH0205832 | ribosomal protein, L38 |
| CH0203745 | transcription factor |
| CH0203746 | transcription factor |
| CH0203748 | transcription factor |
| CH0203750 | transcription factor |
| CH0203763 | transcription factor |
| CH0202335 | Inherit from KOG: Retrotransposon protein |
| CH0213487 | Inherit from KOG: Retrotransposon protein |
| CH0220730 | Inherit from KOG: Retrotransposon protein |
| CH0206419 | Inherit from KOG: Retrotransposon protein |
| CH0201881 | Inherit from KOG: Retrotransposon protein |
| CH0206405 | Inherit from KOG: Retrotransposon protein |
| CH0201054 | Zn-finger in Ran binding protein and others |
| CH0228022 | G-type lectin S-receptor-like serine threonine-protein kinase |
| CH0201055 | serine threonine-protein kinase |
| CH0228025 | serine threonine-protein kinase |
| CH0228026 | serine threonine-protein kinase |
| CH0201077 | serine threonine-protein kinase |
| CH0227625 | NA |
| CH0227648 | NA |
| CH0227652 | Glucosidase, alpha |
| CH0227650 | NA |
| CH0221760 | transposon protein |
| CH0221919 | transposon protein |
| CH0221937 |  |
| CH0227943 | transposon protein |
| CH0221938 | transposon protein |
| CH0204032 | transposon protein |
| CH0218301 | Asp/Glu/Hydantoin racemase |
| CH0218304 | Asp/Glu/Hydantoin racemase |
| CH0218311 | Asp/Glu/Hydantoin racemase |
| CH0218326 | Asp/Glu/Hydantoin racemase |
| CH0228216 | Asp/Glu/Hydantoin racemase |
| CH0222299 | expressed protein |
| CH0222301 | expressed protein |
| CH0222300 | Pentatricopeptide repeat-containing protein |
| CH0222302 | Pentatricopeptide repeat-containing protein |
| CH0213839 | resistance protein |
| CH0213840 | resistance protein |
| CH0213845 |  |
| CH0213853 |  |
| CH0221212 |  |
| CH0221215 |  |
| CH0221217 |  |
| CH0221219 |  |
| CH0221221 |  |
| CH0213043 |  |
| CH0215085 | Retrotransposon protein |
| CH0217315 | Retrotransposon protein |
| CH0221955 |  |
| CH0213630 |  |
| CH0221899 |  |
| CH0213340 |  |
| CH0213358 |  |
| CH0213629 |  |
| CH0221900 |  |
| CH0218385 |  |
| CH0224870 |  |
| CH0213622 | NAC transcription factor |
| CH0213802 | NAC transcription factor |
| CH0213808 |  |
| CH0213813 | NAC transcription factor |
| CH0204511 | POT family |
| CH0204514 | POT family |
| CH0204516 | POT family |
| CH0204518 | POT family |
| CH0204519 | POT family |
| CH0219311 | F-box domain |
| CH0220536 | F-box domain |
| CH0219312 | F-box domain |
| CH0219313 | F-box domain |
| CH0220534 | F-box domain |
| CH0220535 | F-box domain |
| CH0208602 | Phosphatidate cytidylyltransferase family protein |
| CH0208608 | Phosphatidate cytidylyltransferase family protein |
| CH0212259 | CACTA, En Spm sub-class |
| CH0226837 | IQ calmodulin-binding motif family protein |
| CH0226871 | IQ calmodulin-binding motif family protein |
| CH0222194 | Retrotransposon protein |
| CH0222196 | Retrotransposon protein |
| CH0224426 | Retrotransposon protein |
| CH0222203 | Retrotransposon protein |
| CH0225016 | Inherit from KOG: Retrotransposon protein |
| CH0203610 |  |
| CH0203611 |  |
| CH0214392 |  |
| CH0222009 |  |
| CH0206269 | DNA helicase |
| CH0208074 | DNA helicase |
| CH0212147 | Bile acid |
| CH0212157 | Bile acid |
| CH0213471 | Retrotransposon protein |
| CH0224965 | Inherit from KOG: Retrotransposon protein |
| CH0224995 | Retrotransposon protein |
| CH0224966 | Retrotransposon protein |
| CH0224996 | Retrotransposon protein |
| CH0224997 | Retrotransposon protein |
| CH0225002 | Retrotransposon protein |
| CH0213582 | Retrotransposon protein |
| CH0224999 |  |
| CH0225000 |  |
| CH0225003 |  |
| CH0227003 | Solute carrier family 10 (Sodium bile acid cotransporter family), member |
| CH0227012 | Solute carrier family 10 (Sodium bile acid cotransporter family), member |
| CH0200061 | small nuclear ribonucleoprotein |
| CH0200100 | small nuclear ribonucleoprotein |
| CH0204979 | small nuclear ribonucleoprotein |
| CH0226917 | small nuclear ribonucleoprotein |
| CH0212933 | homeobox-leucine zipper protein |
| CH0212944 | homeobox-leucine zipper protein |
| CH0224615 | Inherit from KOG: Retrotransposon protein |
| CH0211919 | NA |
| CH0211920 | NA |
| CH0211931 | NA |
| CH0211932 | NA |
| CH0214511 | NAC domain protein |
| CH0214512 | NAC domain protein |
| CH0214513 | NAC domain |
| CH0214530 | NAC domain protein |
| CH0214537 | NAC domain protein |
| CH0224380 |  |
| CH0224544 |  |
| CH0224540 |  |
| CH0224545 |  |
| CH0224547 |  |
| CH0224383 |  |
| CH0224381 |  |
| CH0224539 |  |
| CH0224382 |  |
| CH0224541 |  |
| CH0224546 |  |
| CH0202720 |  |
| CH0202722 |  |
| CH0202728 |  |
| CH0202721 |  |
| CH0202727 |  |
| CH0221880 | Ribosomal protein |
| CH0221891 | Ribosomal protein |
| CH0203322 |  |
| CH0203323 |  |
| CH0211904 |  |
| CH0203923 |  |
| CH0204615 |  |
| CH0204616 |  |
| CH0203618 |  |
| CH0203624 | Transposase family tnp2 |
| CH0212290 | Transposase family tnp2 |
| CH0212584 |  |
| CH0204111 |  |
| CH0203833 |  |
| CH0220900 | Transposase family tnp2 |
| CH0220901 |  |
| CH0204110 |  |
| CH0203297 | cytochrome P450 |
| CH0203309 | cytochrome P450 |
| CH0203457 | Integrase core domain |
| CH0203458 | Integrase core domain |
| CH0208824 | Integrase core domain |
| CH0208825 | Integrase core domain |
| CH0212318 | Integrase core domain |
| CH0217687 | Integrase core domain |
| CH0217688 | Integrase core domain |
| CH0217689 | Integrase core domain |
| CH0217690 | Integrase core domain |
| CH0220563 | Integrase core domain |
| CH0220834 | Integrase core domain |
| CH0218207 | Inherit from KOG: Retrotransposon protein |
| CH0224486 | Inherit from KOG: Retrotransposon protein |
| CH0204315 | Retrotransposon protein |
| CH0217545 | Retrotransposon protein |
| CH0212226 | NA |
| CH0212227 | NA |
| CH0224309 | NA |
| CH0224300 | NA |
| CH0202622 |  |
| CH0207398 | Cinnamyl alcohol dehydrogenase |
| CH0212825 | Retrotransposon protein |
| CH0222165 |  |
| CH0203101 | Retrotransposon protein |
| CH0204062 | Inherit from KOG: Retrotransposon protein |
| CH0207649 | Inherit from KOG: Retrotransposon protein |
| CH0224011 | Inherit from KOG: Retrotransposon protein |
| CH0227001 | Inherit from KOG: Retrotransposon protein |
| CH0210387 | Inherit from KOG: Retrotransposon protein |
| CH0204531 |  |
| CH0212174 |  |
| CH0213765 |  |
| CH0213518 |  |
| CH0213554 |  |
| CH0207684 | MULE transposase domain |
| CH0207709 | MULE transposase domain |
| CH0207912 |  |
| CH0207929 |  |
| CH0212568 |  |
| CH0212572 |  |
| CH0227480 |  |
| CH0217206 |  |
| CH0227478 |  |
| CH0217230 |  |
| CH0217231 |  |
| CH0227479 |  |
| CH0222193 | Retrotransposon protein |
| CH0224425 | Retrotransposon protein |
| CH0203676 |  |
| CH0224709 |  |
| CH0212495 |  |
| CH0212510 |  |
| CH0205131 |  |
| CH0210695 |  |
| CH0226481 | subtilase family |
| CH0226564 | subtilase family |
| CH0226541 | ABC transporter C family member 10-like |
| CH0207614 |  |
| CH0211547 |  |
| CH0213332 |  |
| CH0209561 |  |
| CH0209559 |  |
| CH0205698 | smt3 suppressor of mif two 3 homolog |
| CH0205793 | smt3 suppressor of mif two 3 homolog |
| CH0207598 |  |
| CH0207599 |  |
| CH0208929 |  |
| CH0209647 |  |
| CH0208934 |  |
| CH0209644 |  |
| CH0209645 |  |
| CH0209646 |  |
| CH0208935 |  |
| CH0213637 | DNA-binding protein |
| CH0213725 | DNA-binding protein |
| CH0218412 | F-box kelch-repeat protein At3g06240-like |
| CH0218535 |  |
| CH0218536 | F-box kelch-repeat protein At3g06240-like |
| CH0218538 | F-box kelch-repeat protein At3g06240-like |
| CH0221358 |  |
| CH0221365 |  |
| CH0221360 |  |
| CH0221366 |  |
| CH0204129 | CHROMO |
| CH0228101 | CHROMO |
| CH0208457 |  |
| CH0217366 |  |
| CH0217369 |  |
| CH0200355 | Quinone oxidoreductase-like protein At1g23740 |
| CH0225211 | Quinone oxidoreductase-like protein At1g23740 |
| CH0200360 | Quinone oxidoreductase-like protein At1g23740 |
| CH0202674 | Quinone oxidoreductase-like protein At1g23740 |
| CH0220106 | Quinone oxidoreductase-like protein At1g23740 |
| CH0224317 | Quinone oxidoreductase-like protein At1g23740 |
| CH0200492 | resistance protein |
| CH0200515 | resistance protein |
| CH0200351 | resistance protein |
| CH0203024 |  |
| CH0217904 | protein FAR1-RELATED SEQUENCE |
| CH0207785 |  |
| CH0223094 |  |
| CH0211945 |  |
| CH0213055 |  |
| CH0217160 |  |
| CH0207930 |  |
| CH0208010 |  |
| CH0208011 |  |
| CH0208099 |  |
| CH0228001 |  |
| CH0228004 |  |
| CH0228295 |  |
| CH0210805 |  |
| CH0222016 | Inherit from KOG: Retrotransposon protein |
| CH0213848 |  |
| CH0213849 |  |
| CH0217707 |  |
| CH0219315 | STYKc |
| CH0220520 | STYKc |
| CH0220524 | STYKc |
| CH0220526 | STYKc |
| CH0220530 | STYKc |
| CH0228039 | NA |
| CH0228040 | NA |
| CH0228042 | NA |
| CH0228043 | NA |
| CH0228064 | NA |
| CH0228090 | NA |
| CH0203714 |  |
| CH0207698 |  |
| CH0217871 |  |
| CH0211947 |  |
| CH0212468 |  |
| CH0203887 | LRR receptor-like serine threonine-protein kinase |
| CH0208310 | LRR receptor-like serine threonine-protein kinase |
| CH0208320 | receptor-like protein kinase |
| CH0203995 | receptor-like protein |
| CH0204012 | Ribosomal protein L12 |
| CH0204180 |  |
| CH0204185 |  |
| CH0204196 |  |
| CH0228017 |  |
| CH0204181 |  |
| CH0204845 |  |
| CH0204857 |  |
| CH0216855 |  |
| CH0216870 |  |
| CH0205687 | NB-ARC domain |
| CH0205795 | NB-ARC domain |
| CH0205798 | NB-ARC domain |
| CH0205692 | NB-ARC domain |
| CH0207934 | calcineurin |
| CH0208676 |  |
| CH0207936 |  |
| CH0208289 | transposon protein |
| CH0212569 | transposon protein |
| CH0218314 | transposon protein |
| CH0221607 | transposon protein |
| CH0211754 | transposon protein |
| CH0211607 | Inherit from KOG: Retrotransposon protein |
| CH0228246 | Inherit from KOG: Retrotransposon protein |
| CH0217752 | Inherit from KOG: Retrotransposon protein |
| CH0213036 |  |
| CH0213041 |  |
| CH0213627 |  |
| CH0224863 |  |
| CH0217011 |  |
| CH0217497 |  |
| CH0220521 | STYKc |
| CH0220527 | STYKc |
| CH0200226 |  |
| CH0200232 |  |
| CH0204178 | NB-ARC domain |
| CH0204186 | NB-ARC domain |
| CH0228015 | NB-ARC domain |
| CH0204205 | NB-ARC domain |
| CH0208009 | Catalyzes the S-methylmethionine (SMM) biosynthesis from adenosyl-L-homocysteine (AdoMet) and methionine. SMM biosynthesis (by MMT1) and degradation (by HMT-1, HMT-2 and HMT-3) constitute the SMM cycle in plants, which is probably required to achieve short term control of AdoMet level. Also able to catalyze the selenium-methylmethionine (SeMM) from AdoMet and selenium- methionine (SeMet). May play a role in phoem sulfur transport |
| CH0227999 | Catalyzes the S-methylmethionine (SMM) biosynthesis from adenosyl-L-homocysteine (AdoMet) and methionine. SMM biosynthesis (by MMT1) and degradation (by HMT-1, HMT-2 and HMT-3) constitute the SMM cycle in plants, which is probably required to achieve short term control of AdoMet level. Also able to catalyze the selenium-methylmethionine (SeMM) from AdoMet and selenium- methionine (SeMet). May play a role in phoem sulfur transport |
| CH0228002 | Catalyzes the S-methylmethionine (SMM) biosynthesis from adenosyl-L-homocysteine (AdoMet) and methionine. SMM biosynthesis (by MMT1) and degradation (by HMT-1, HMT-2 and HMT-3) constitute the SMM cycle in plants, which is probably required to achieve short term control of AdoMet level. Also able to catalyze the selenium-methylmethionine (SeMM) from AdoMet and selenium- methionine (SeMet). May play a role in phoem sulfur transport |
| CH0228005 | Catalyzes the S-methylmethionine (SMM) biosynthesis from adenosyl-L-homocysteine (AdoMet) and methionine. SMM biosynthesis (by MMT1) and degradation (by HMT-1, HMT-2 and HMT-3) constitute the SMM cycle in plants, which is probably required to achieve short term control of AdoMet level. Also able to catalyze the selenium-methylmethionine (SeMM) from AdoMet and selenium- methionine (SeMet). May play a role in phoem sulfur transport |
| CH0208269 |  |
| CH0208283 |  |
| CH0208270 |  |
| CH0208282 |  |
| CH0212049 | cyanidin-3-O-glucoside 2-O-glucuronosyltransferase-like |
| CH0212050 | cyanidin-3-O-glucoside 2-O-glucuronosyltransferase-like |
| CH0212059 | cyanidin-3-O-glucoside 2-O-glucuronosyltransferase-like |
| CH0212058 | cyanidin-3-O-glucoside 2-O-glucuronosyltransferase-like |
| CH0213035 |  |
| CH0213042 |  |
| CH0213628 |  |
| CH0221954 |  |
| CH0222964 | flavin-containing monooxygenase |
| CH0222988 | flavin-containing monooxygenase |
| CH0222971 | flavin-containing monooxygenase |
| CH0223852 | Pentatricopeptide repeat-containing protein |
| CH0223867 | Pentatricopeptide repeat-containing protein |
| CH0223899 | Pentatricopeptide repeat-containing protein |
| CH0224305 |  |
| CH0224315 |  |
| CH0224316 |  |
| CH0224325 |  |
| CH0225190 | LRR receptor-like serine threonine-protein kinase |
| CH0228065 | LRR receptor-like serine threonine-protein kinase |
| CH0228067 | LRR receptor-like serine threonine-protein kinase |
| CH0228072 | LRR receptor-like serine threonine-protein kinase |
| CH0202774 |  |
| CH0224753 |  |
| CH0212968 |  |
| CH0203711 | Endonuclease/Exonuclease/phosphatase family |
| CH0214700 | Retrotransposon protein |
| CH0214604 | Endonuclease/Exonuclease/phosphatase family |
| CH0204149 | NB-ARC domain |
| CH0207831 | NB-ARC domain |
| CH0204208 | NB-ARC domain |
| CH0204184 | resistance protein |
| CH0228019 | resistance protein |
| CH0204206 | resistance protein |
| CH0204187 | resistance protein |
| CH0204197 | resistance protein |
| CH0228016 | resistance protein |
| CH0209481 | G-type lectin S-receptor-like serine threonine-protein kinase |
| CH0209488 | G-type lectin S-receptor-like serine threonine-protein kinase |
| CH0209483 | G-type lectin S-receptor-like serine threonine-protein kinase |
| CH0212100 |  |
| CH0224642 | NA |
| CH0217786 |  |
| CH0212196 | NADP-dependent D-sorbitol-6-phosphate |
| CH0212201 | NADP-dependent D-sorbitol-6-phosphate |
| CH0212299 | NADP-dependent D-sorbitol-6-phosphate |
| CH0212197 | Control of topological states of DNA by transient breakage and subsequent rejoining of DNA strands. Topoisomerase II makes double-strand breaks |
| CH0212200 | Control of topological states of DNA by transient breakage and subsequent rejoining of DNA strands. Topoisomerase II makes double-strand breaks |
| CH0212300 | Control of topological states of DNA by transient breakage and subsequent rejoining of DNA strands. Topoisomerase II makes double-strand breaks |
| CH0212489 | cysteine) proteinase inhibitor |
| CH0212490 | cysteine) proteinase inhibitor |
| CH0212491 | cytochrome P450 |
| CH0212697 | resistance protein |
| CH0212707 | resistance protein |
| CH0212711 | resistance protein |
| CH0212703 | FAS1 |
| CH0212761 | methyltransferase like 13 |
| CH0217476 | FAS1 |
| CH0212704 |  |
| CH0212760 |  |
| CH0212705 |  |
| CH0212903 |  |
| CH0224754 |  |
| CH0225118 |  |
| CH0218305 |  |
| CH0228217 |  |
| CH0218327 |  |
| CH0203806 |  |
| CH0203813 |  |
| CH0203857 |  |
| CH0227060 | Protein FAR-RED IMPAIRED RESPONSE |
| CH0203936 |  |
| CH0213223 |  |
| CH0204045 | Protein FAR-RED IMPAIRED RESPONSE |
| CH0217895 | protein FAR1-RELATED SEQUENCE |
| CH0204059 | Inherit from KOG: Retrotransposon protein |
| CH0207821 | Inherit from KOG: Retrotransposon protein |
| CH0204175 |  |
| CH0204183 |  |
| CH0204530 |  |
| CH0204539 |  |
| CH0205065 |  |
| CH0205097 |  |
| CH0207600 |  |
| CH0224302 |  |
| CH0207647 |  |
| CH0221948 | Multicopper oxidase |
| CH0207719 | AHA1, activator of heat shock 90kDa protein ATPase homolog |
| CH0218515 | AHA1, activator of heat shock 90kDa protein ATPase homolog |
| CH0208158 |  |
| CH0221757 |  |
| CH0208751 | DNA repair protein rad51 |
| CH0208753 | DNA repair protein rad51 |
| CH0211620 |  |
| CH0218259 |  |
| CH0211816 | Retrotransposon protein |
| CH0211832 | Retrotransposon protein |
| CH0211840 | B3 domain-containing protein |
| CH0211842 | B3 domain-containing protein |
| CH0212444 | NA |
| CH0212445 | NA |
| CH0212545 |  |
| CH0216708 |  |
| CH0213038 |  |
| CH0213039 |  |
| CH0213519 | zinc finger CCCH domain-containing protein |
| CH0213931 | zinc finger CCCH domain-containing protein |
| CH0213671 | Glucosidase, alpha |
| CH0214860 |  |
| CH0215069 |  |
| CH0215070 |  |
| CH0215999 | DUF246 domain-containing protein |
| CH0227990 | DUF246 domain-containing protein |
| CH0216856 |  |
| CH0216862 | Patatin group |
| CH0216882 | Histone-lysine N-methyltransferase |
| CH0216883 |  |
| CH0218502 |  |
| CH0218505 |  |
| CH0219246 |  |
| CH0228086 |  |
| CH0219638 | UDP-Glycosyltransferase |
| CH0219651 | UDP-Glycosyltransferase |
| CH0221689 | Zinc finger (C3HC4-type RING finger) family protein |
| CH0221690 | Zinc finger (C3HC4-type RING finger) family protein |
| CH0222173 |  |
| CH0222197 |  |
| CH0224333 |  |
| CH0224352 |  |
| CH0224737 | Mur ligase middle domain |
| CH0224739 |  |
| CH0224768 |  |
| CH0224769 |  |
| CH0226789 | ubiquitin-conjugating enzyme |
| CH0226803 | ubiquitin-conjugating enzyme |
| CH0227713 | diacylglycerol kinase |
| CH0228033 | diacylglycerol kinase |
| CH0228041 | Core subunit of the mitochondrial membrane respiratory chain NADH dehydrogenase (Complex I) that is believed to belong to the minimal assembly required for catalysis. Complex I functions in the transfer of electrons from NADH to the respiratory chain. The immediate electron acceptor for the enzyme is believed to be ubiquinone |
| CH0228044 | Core subunit of the mitochondrial membrane respiratory chain NADH dehydrogenase (Complex I) that is believed to belong to the minimal assembly required for catalysis. Complex I functions in the transfer of electrons from NADH to the respiratory chain. The immediate electron acceptor for the enzyme is believed to be ubiquinone |

**Table S9 Go analysis of DEGs of *C. humilis* under drought stress**

| GO.ID | Term | Annotated | Significant | Expected | classicFisher | FDR |
| --- | --- | --- | --- | --- | --- | --- |
| GO:0015979 | photosynthesis | 124 | 78 | 32.64 | 7.00E-18 | 3.95E-14 |
| GO:0019684 | photosynthesis, light reaction | 89 | 58 | 23.42 | 1.30E-14 | 3.66E-11 |
| GO:0055114 | oxidation-reduction process | 627 | 235 | 165.02 | 8.90E-11 | 1.67E-07 |
| GO:0006091 | generation of precursor metabolites and ... | 174 | 83 | 45.8 | 7.60E-10 | 1.07E-06 |
| GO:0009773 | photosynthetic electron transport in pho... | 16 | 15 | 4.21 | 2.30E-08 | 2.59E-05 |
| GO:0080167 | response to karrikin | 136 | 63 | 35.79 | 3.30E-07 | 0.00031 |
| GO:0009767 | photosynthetic electron transport chain | 52 | 31 | 13.69 | 3.90E-07 | 0.0003141 |
| GO:0022900 | electron transport chain | 64 | 35 | 16.84 | 1.30E-06 | 0.000916 |
| GO:0009657 | plastid organization | 153 | 64 | 40.27 | 1.80E-05 | 0.011274 |
| GO:0009765 | photosynthesis, light harvesting | 15 | 12 | 3.95 | 2.10E-05 | 0.0118377 |
| GO:0009314 | response to radiation | 528 | 178 | 138.97 | 5.50E-05 | 0.028185 |
| GO:0033013 | tetrapyrrole metabolic process | 52 | 27 | 13.69 | 7.00E-05 | 0.0328825 |
| GO:0009416 | response to light stimulus | 514 | 173 | 135.28 | 7.80E-05 | 0.033822 |
| GO:0009269 | response to desiccation | 7 | 7 | 1.84 | 8.70E-05 | 0.0350299 |
