# Supplementary material for: The genome of Prunus humilis provides new insights to drought adaption and population diversity
Source: DNA Res. 2022 Jun 25;29(4):dsac021. doi: 10.1093/dnares/dsac021 (PMC9278622; doi:10.1093/dnares/dsac021)

**Figure S1 Genome size and heterozygosity analysis based on k-mer analysis (K=17)**


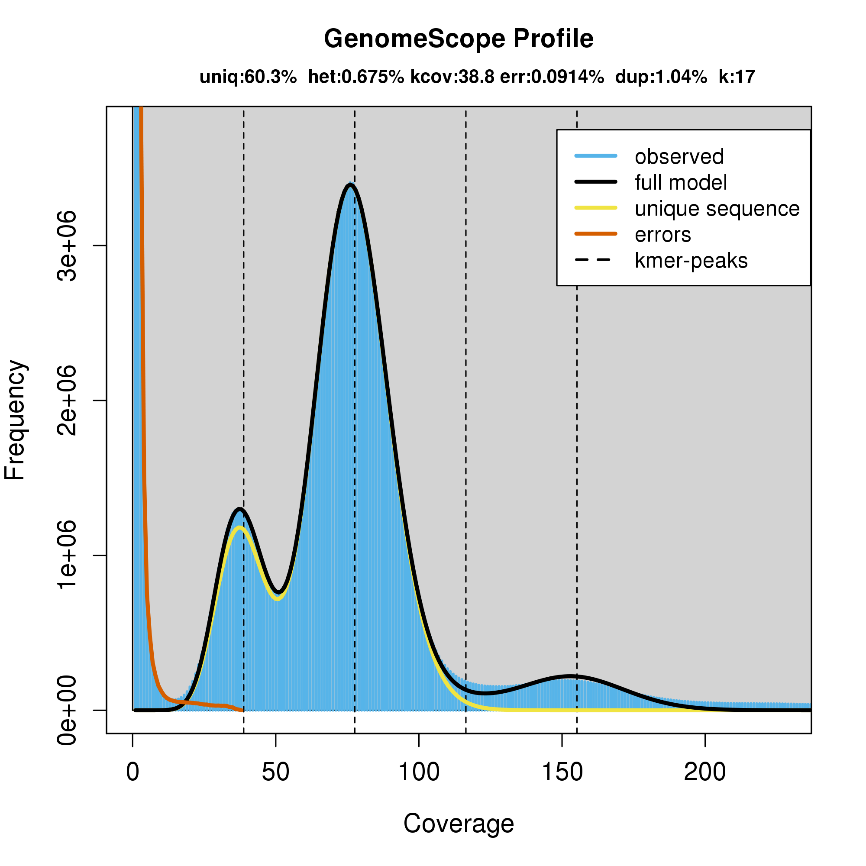


**Figure S2 Hi-C interactive heatmap of the** ***C .humilis*. the x-axis and y-axis is the genome of *C .humilis*, each blue box indicate a super-scaffold.**


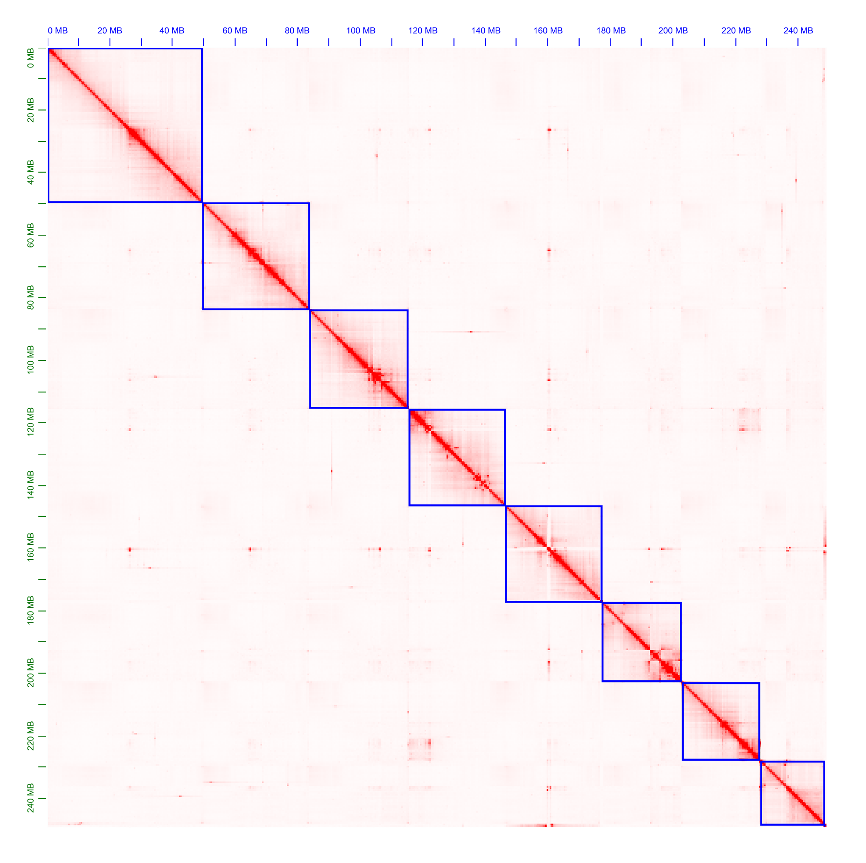


**Figure S3 Density and distribution of four type small RNAs on 8 chromosomes of *C .humilis***


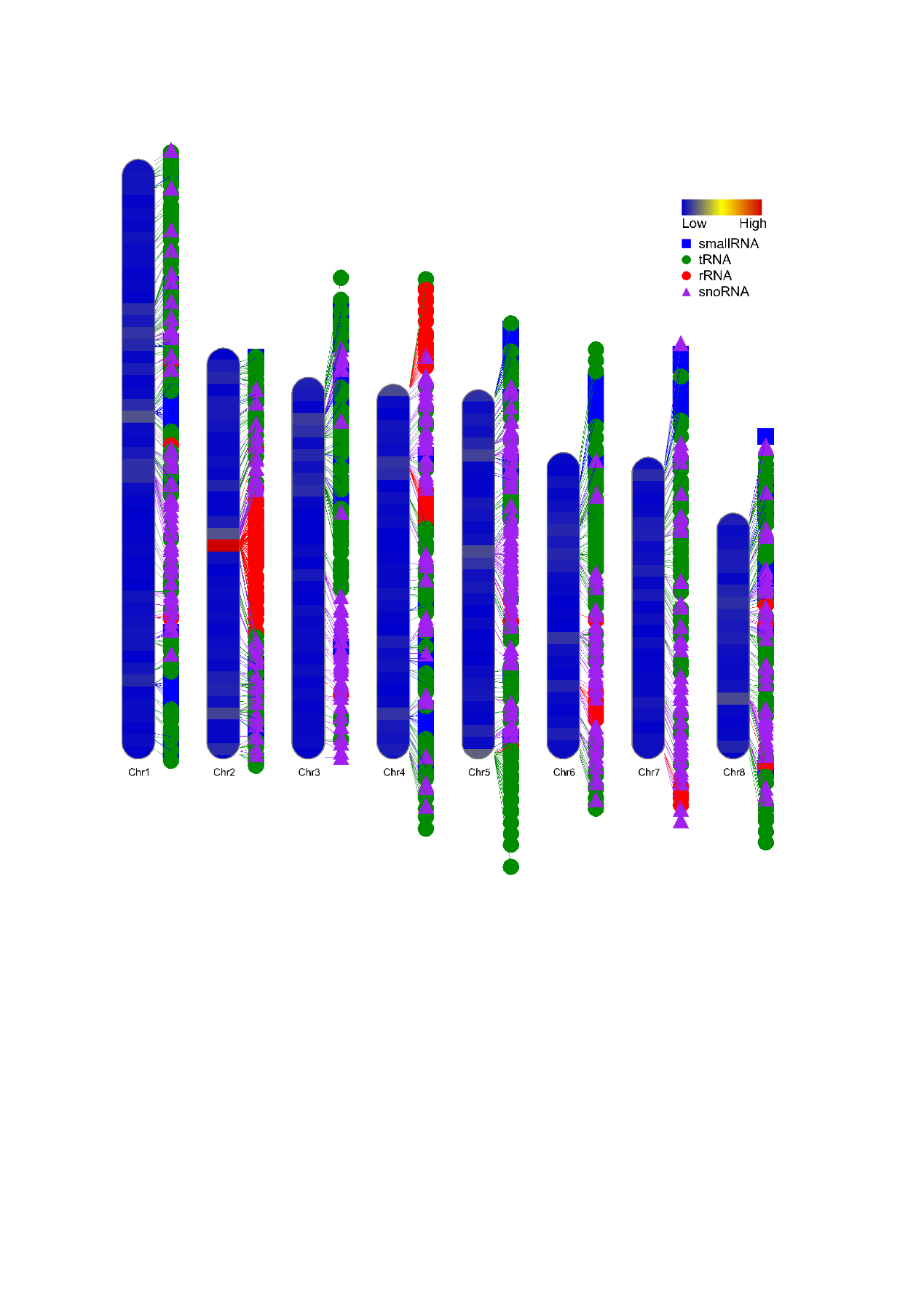


**Figure S4** **Density and distribution of rRNAs on 8 chromosomes of** ***C .humilis***


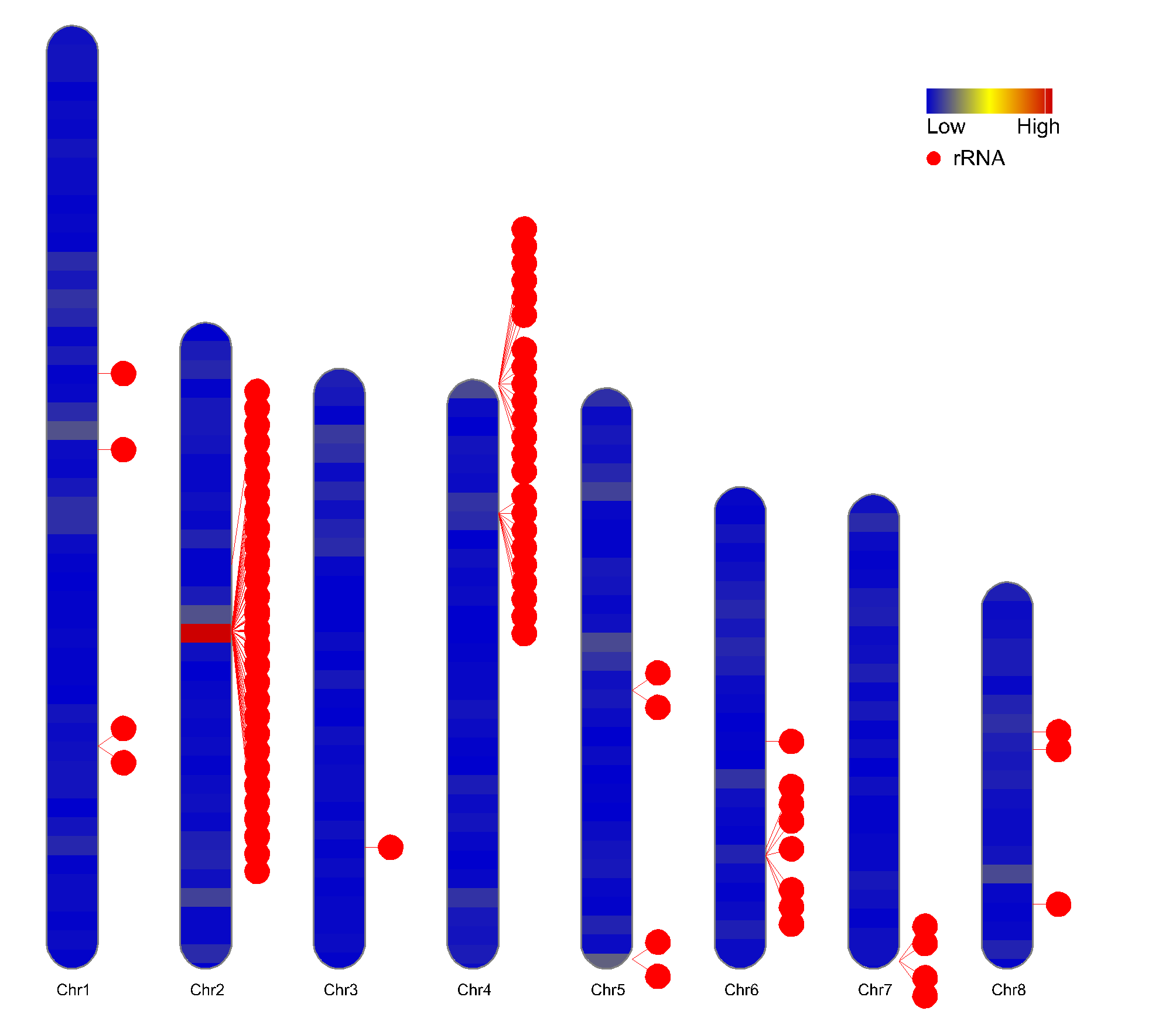


**Figure S5 Statistics of the annotated protein coding genes in different database**


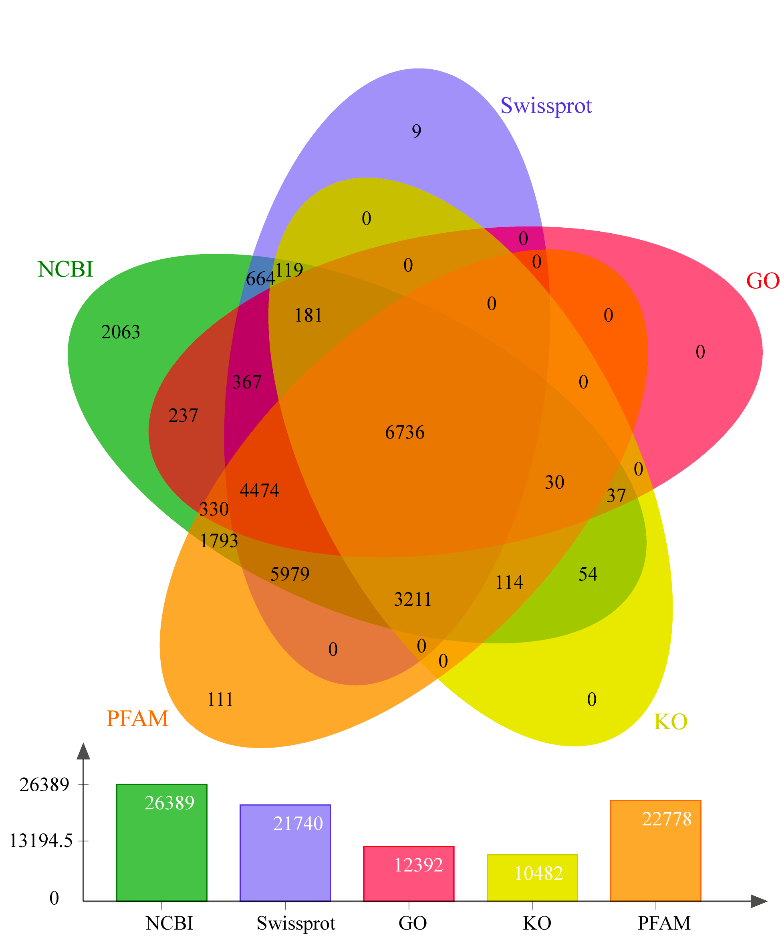


**Figure S6 The evolutionary relationship of *Prunus***


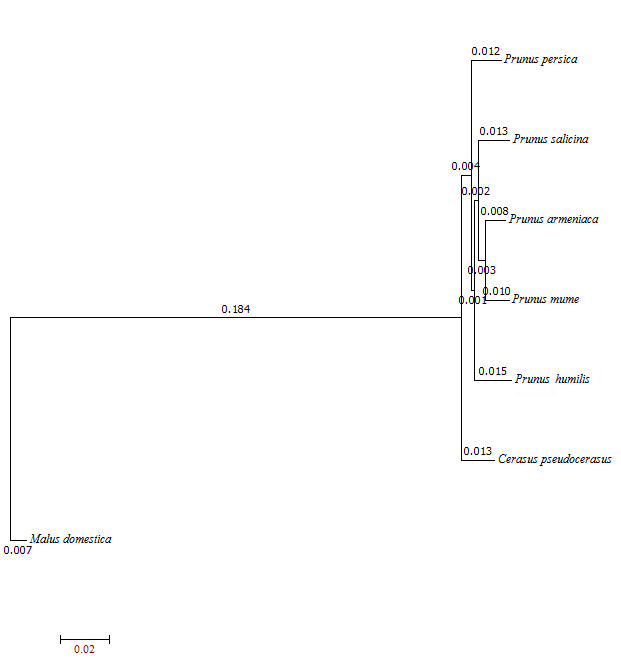


**Figure S7 Ks analysis of *LEA* genes involved segment duplication in *C .humilis***


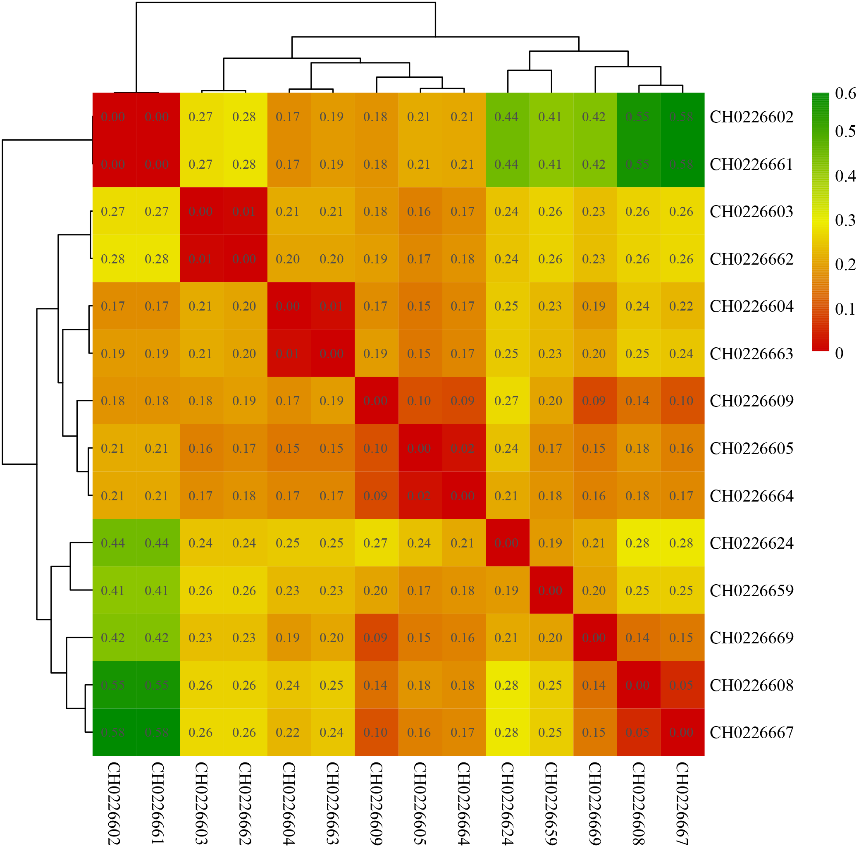

Supplement: dsac021_Supplementary_Data [file dsac021_supplementary_data.zip › S Figure.docx]
